# Supplementary material for: Acceleration, simplification and potential parallelization of digital polymers sequencing by coupling tandem mass spectrometry with ion mobility
Source: Nat Commun. 2025 Nov 26;16:11642. doi: 10.1038/s41467-025-66762-0 (PMC12748738; doi:10.1038/s41467-025-66762-0)
Supplement: Supplementary file 1 — Supplementary information [file 41467_2025_66762_MOESM1_ESM.pdf]

Supplementary Information for

Acceleration, simplification and potential parallelization of digital polymers sequencing by coupling tandem mass spectrometry with ion mobility

Isaure Sergent,[1] Georgette Obeid,[2] Thibault Schutz,[2] Jean-François Lutz,[2]* Laurence Charles[1]*

[1] Aix Marseille Université, CNRS, UMR 7273, Institut de Chimie Radicalaire (ICR), 13397 Marseille Cedex 20, France

[2] Université de Strasbourg, CNRS, Institut de Science et d'Ingénierie Supramoléculaires (ISIS), 67000 Strasbourg, France

content	page
Supplementary Table 1. List of the b-PPDE triblocks used as models ………………………..	S2
Supplementary Fig. 1. Selection of precursor ion charge state ………………………………..	S3
Supplementary Fig. 2. Arrival times independent of 0/1 sequence …………………………...	S4
Supplementary Fig. 3. Optimization of TWIMS conditions …………………………………..	S4
Supplementary Table 2. Prediction of block t_A (ms) in IMS ………………………………….	S5
Supplementary Fig. 4. Structure of b-PPDEs coding for ICR and PHew! ………………..	S5
Supplementary Fig. 5. Sequencing of the three blocks of the "ICR" polymer ………….	S6
Supplementary Table 3. Sequencing fragments of B1 in the "ICR" polymer ………………...	S7
Supplementary Table 4. Sequencing fragments of B2 in the "ICR" polymer ………………...	S8
Supplementary Table 5. Sequencing fragments of B3 in the "ICR" polymer ………………...	S9
Supplementary Table 6. The ASCII code for letters of the Latin alphabet ……………………	S10
Supplementary Fig. 6. IMS traces extracted for blocks of the "PHew!" polymer …………….	S10
Supplementary Fig. 7. Sequencing of the five blocks of the "PHew!" polymer ………………	S11
Supplementary Table 7. Sequencing fragments of B1 in the "PHew!" polymer ……………...	S12
Supplementary Table 8. Sequencing fragments of B2 in the "PHew!" polymer ……………...	S13
Supplementary Table 9. Sequencing fragments of B3 in the "PHew!" polymer ……………...	S14
Supplementary Table 10. Sequencing fragments of B4 in the "PHew!" polymer ……….…...	S15
Supplementary Table 11. Sequencing fragments of B5 in the "PHew!" polymer …………….	S16
Supplementary Fig. 8. Predicted t_A ranges for singly charged Bi in MALDI …………………	S17
Supplementary Table 12. Sequencing fragments of B1 in "ICR" by MALDI ………………..	S18
Supplementary Table 13. Sequencing fragments of B2 in "ICR" by MALDI ………………..	S19
Supplementary Table 14. Sequencing fragments of B3 in "ICR" by MALDI ………………..	S20
Supplementary protocol: de novo sequencing ………………………………………………...	S21

	full sequence	$[B1 - 2H]^{2-}$	$[B2 - 2H]^{2-}$	$[B3 - 2H]^{2-}$
P1	0101000-T5-01001101-T6-01000011	$0_6 1_2$ *m/z* 653.6	T5-$0_4 1_4$ *m/z* 910.2	T6-$0_5 1_3$ *m/z* 777.7
P2	10101010-T2-10101010-T8-10101010	$0_4 1_4$ *m/z* 681.6	T2-$0_4 1_4$ *m/z* 872.2	T8-$0_4 1_4$ *m/z* 834.7
P3	01010000-T3-01001101-T2-01000011	$0_6 1_2$ *m/z* 653.6	T3-$0_4 1_4$ *m/z* 881.2	T2-$0_5 1_3$ *m/z* 752.7
P4	11000010-T6-10110010-T8-00001010	$0_5 1_3$ *m/z* 667.6	T6-$0_4 1_4$ *m/z* 897.2	T8-$0_6 1_2$ *m/z* 806.7
P5	01010000-T8-01001101-T6-01000011	$0_6 1_2$ *m/z* 653.6	T8-$0_4 1_4$ *m/z* 940.2	T6-$0_5 1_3$ *m/z* 777.7
P6	11111111-T9-00000000-T8-111111111	$0_0 1_8$ *m/z* 737.7	T9-$0_8 1_0$ *m/z* 844.2	T8-$0_0 1_8$ *m/z* 890.8
P7	11111111-T2-00000000-T3-111111111	$0_0 1_8$ *m/z* 737.7	T2-$0_8 1_0$ *m/z* 816.2	T3-$0_0 1_8$ *m/z* 831.8
P8	00000000-T2-11111111-T8-00000000	$0_8 1_0$ *m/z* 625.6	T2-$0_0 1_8$ *m/z* 928.3	T8-$0_8 1_0$ *m/z* 778.6
P9	01010010-T3-01000011-T2-01010011	$0_5 1_3$ *m/z* 667.6	T3-$0_5 1_3$ *m/z* 867.2	T2-$0_4 1_4$ *m/z* 766.7
P10	01010000-T5-01001101-T9-01000011	$0_6 1_2$ *m/z* 653.6	T5-$0_4 1_4$ *m/z* 910.3	T9-$0_5 1_3$ *m/z* 780.7
P11	11000010-T6-10110010-T5-00001010	$0_5 1_3$ *m/z* 667.6	T6-$0_4 1_4$ *m/z* 897.3	T5-$0_6 1_2$ *m/z* 776.7

Supplementary Table 1. List of the b-PPDE triblocks used as models for this study, with co-monomeric composition and *m/z* values of individual blocks at charge state 2−.

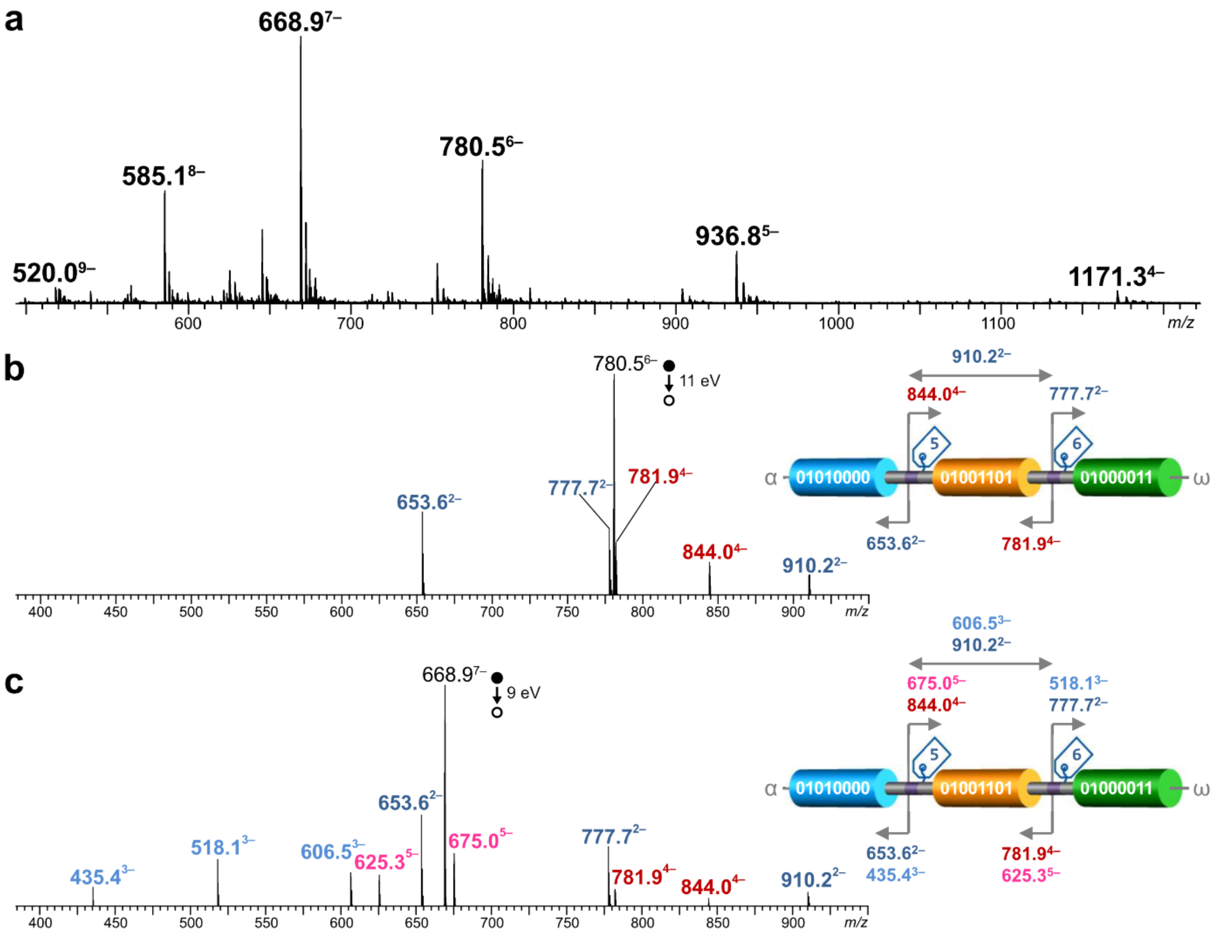

Supplementary Fig. 1. Selection of precursor ion charge state. **a** In negative mode ESI-MS of b-PPDE with n bytes, most stable species are always observed with (3n – 2) charges (n = 3 ↔ major signal for $[M - 7H]^{7-}$). However, in spite of its slightly lower abundance, selecting $[M - 6H]^{6-}$ at *m/z* 780.5 as the precursor ion for MS/MS is best at preventing signal dilution because, thanks to even distribution of the six charges over the three blocks, each fragment is produced at a single charge state as shown in **b**. In contrast, the odd number of charges in $[M - 7H]^{7-}$ at *m/z* 668.9 leads to production of each fragment at two different charge states as shown in **c**. Accordingly, in order to guarantee detectability of sequencing fragments in MS^3 experiments, primary precursor ions containing n blocks need to be selected with z = 2n in order to ensure exclusive production of secondary precursor ions as $[Bi - 2H]^{2-}$ anions.

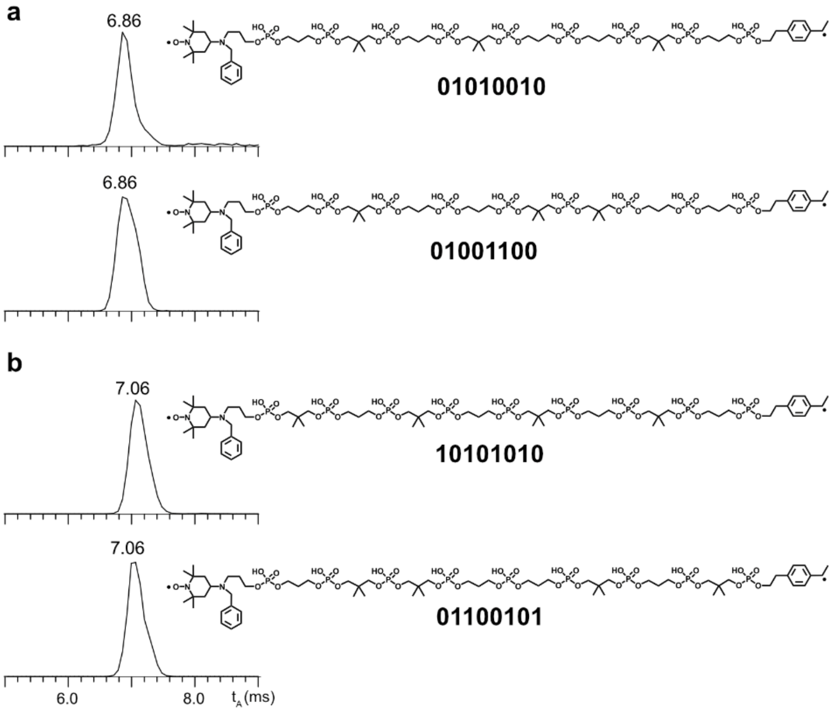

Supplementary Fig. 2. Arrival times independent of 0/1 sequence. The same arrival times (t_A in ms) are measured for blocks of the same category, with the same tag and the same co-monomeric composition, regardless of their sequence. This is exemplified here with **a** the same t_A = 6.86 ms measured for inner blocks with tag T2 containing 5 bits 0 and 3 bits 1 distributed according to different sequences, 01010010 (top) or 01001100 (bottom); **b** the same t_A = 7.06 ms measured for inner blocks with tag T2 containing 4 bits 0 and 4 bits 1 distributed according to different sequences, 10101010 (top) or 01100101 (bottom).

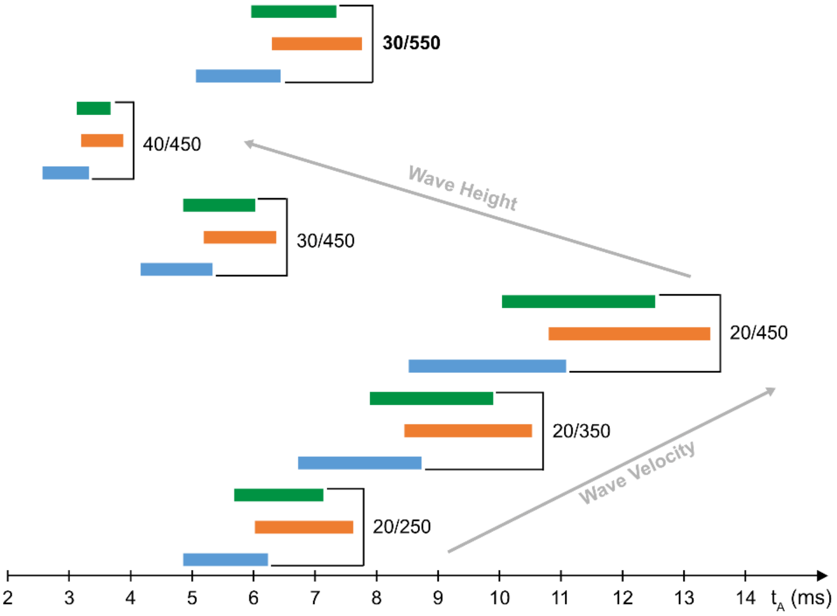

Supplementary Fig. 3. Optimization of TWIMS conditions. Variation of t_A ranges of doubly deprotonated blocks (first: blue, inner: orange, last: green) as a function of WH (in V)/WV (in m.s^{-1}) experimental conditions used to operate the TWIM cell. Best compromise between low t_A values targeted for first and last blocks and optimal block resolution was found with WH = 30 V and WV = 550 m.s^{-1} (shown on top).

	0_81_0	0_71_1	0_61_2	0_51_3	0_41_4	0_31_5	0_21_6	0_11_7	0_01_8	predictive Eq.
**First block**										
-	**5.06**	5.22	**5.40**	**5.58**	**5.78**	5.94	6.12	6.30	**6.48**	y=0.178x+5.05
**Inner block**										
	0_81_0	0_71_1	0_61_2	0_51_3	0_41_4	0_31_5	0_21_6	0_11_7	0_01_8	predictive Eq.
T2	**6.34**	6.53	6.71	6.88	**7.06**	7.23	7.41	7.58	**7.76**	y=0.178x+6.34
T3	6.34	6.53	6.71	**6.88**	**7.06**	7.23	7.41	7.58	7.76	y=0.178x+6.34
T5	6.88	7.05	7.23	7.41	**7.59**	7.76	7.94	8.12	8.29	y=0.178x+6.88
T6	6.60	6.77	6.95	7.13	**7.31**	7.48	7.66	7.84	8.01	y=0.178x+6.60
T8	6.63	6.81	6.99	7.16	**7.34**	7.52	7.69	7.87	8.05	y=0.178x+6.63
T9	**6.72**	6.88	7.06	7.23	**7.41**	7.59	7.76	7.94	8.12	y=0.178x+6.72
**Last block**										
	0_81_0	0_71_1	0_61_2	0_51_3	0_41_4	0_31_5	0_21_6	0_11_7	0_01_8	predictive Eq.
T2	5.63	5.81	5.98	**6.16**	**6.37**	6.55	6.72	6.90	7.08	y=0.178x+5.63
T3	5.71	5.89	6.07	6.25	6.42	6.60	6.78	6.95	**7.13**	y=0.178x+5.71
T5	6.08	6.26	**6.44**	6.62	6.79	6.97	7.15	7.33	7.50	y=0.178x+6.08
T6	5.87	6.05	6.23	**6.41**	6.58	6.76	6.94	7.11	7.29	y=0.178x+5.87
T8	**5.99**	6.20	**6.37**	6.55	**6.72**	6.87	7.06	7.23	**7.41**	y=0.178x+5.99
T9	5.98	6.16	6.33	**6.51**	6.69	6.86	7.04	7.22	7.40	y=0.178x+5.98

Supplementary Table 2. Prediction of block t_A (ms) as a function of their category (first: blue; inner: orange; last: green), Ti tag (first column) and co-monomeric composition based on the predictive equation shown in the last column (with y standing for t_A and x, the number of 1-bits in the block). Experimentally measured t_A values are in bold (WV = 30 V, WH = 550 m.s^{-1}). For any given "category/tag" combination, measured t_A values are observed to increase linearly with the number of 1-bits but the slope of this linear trend is constant and equal to 0.178, regardless of the category and tag. Accordingly, for each "category/tag" combination, the equation of this line was established either experimentally when multiple samples were available or calculated from a single measurement.

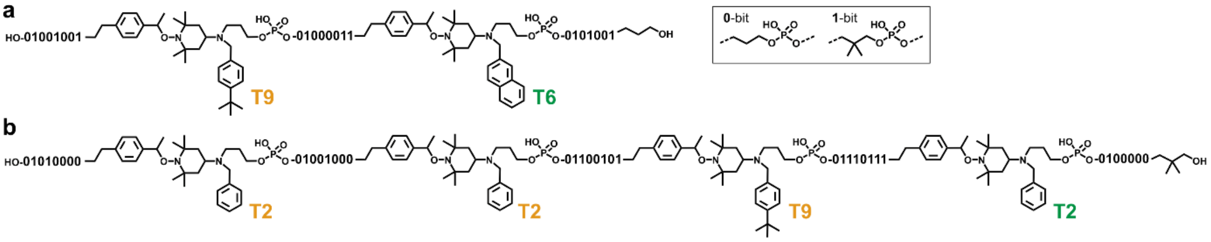

Supplementary Fig. 4. Structure of b-PPDEs coding for **a** "ICR" and **b** "PHew!". Monomers respectively coding for 0 and 1 are shown in inset. The color used for tag names designates the category of block they label (inner: orange, last: green).

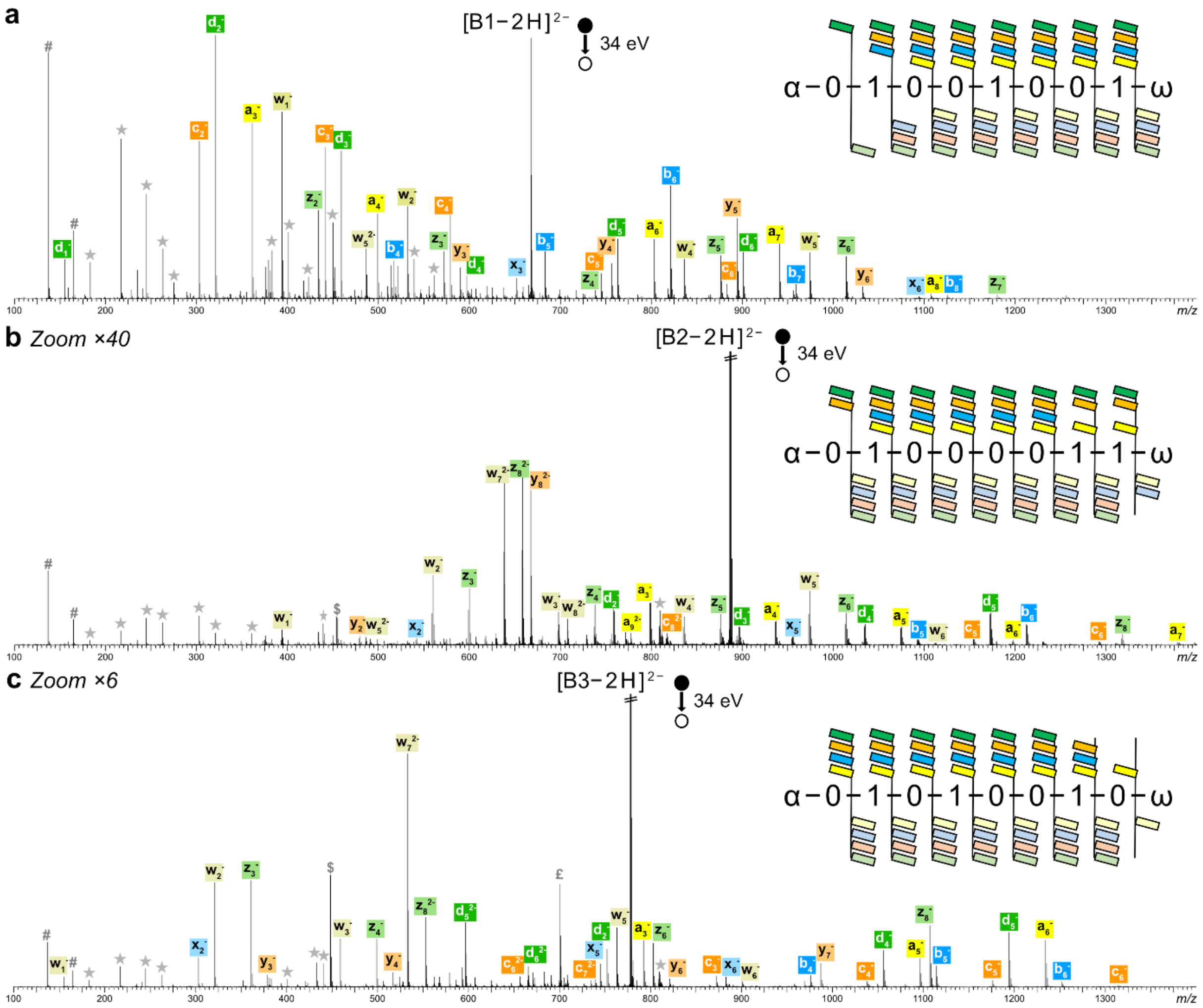

Supplementary Fig. 5. Reading "ICR" coded in a PPDE triblock. Sequencing of the three blocks of the "ICR" b-PPDE from CID spectra recorded for **a** $[B1 - 2H]^{2-}$ at *m/z* 667.6, **b** $[B2 - 2H]^{2-}$ at *m/z* 886.2 and **c** $[B3 - 2H]^{2-}$ at *m/z* 777.7, with inset schemes showing full sequence coverage. Grey symbols: $: tag; #: deprotonated monomer; £: loss of TEMPO; stars: internal fragments. Accurate mass measurements of sequencing fragments are reported in Supplementary Tables 3-5.

a_i^{z-}<br>m/z_{th}<br>m/z_{exp}	*n.e.*	*n.e.*	$C_{11}H_{23}O_9P_2^-$<br>361.0823<br>361.0833	$C_{14}H_{30}O_{13}P_3^-$<br>499.0905<br>499.0922	$C_{19}H_{41}O_{17}P_4^-$<br>665.1300<br>665.1298	$C_{22}H_{48}O_{21}P_5^-$<br>803.1382<br>803.1385	$C_{25}H_{55}O_{25}P_6^-$<br>941.1464<br>941.1474	$C_{30}H_{66}O_{29}P_7^-$<br>1107.1859<br>1107.1804	
b_i^{z-}<br>m/z_{th}<br>m/z_{exp}	*n.e.*	$C_8H_{18}O_6P^-$<br>241.0847<br>241.0860	$C_{11}H_{25}O_{10}P_2^-$<br>379.0928<br>379.0944	$C_{14}H_{32}O_{14}P_3^-$<br>517.1010<br>517.1024	$C_{19}H_{43}O_{18}P_4^-$<br>683.1405<br>683.1410	$C_{22}H_{50}O_{22}P_5^-$<br>821.1487<br>821.1494	$C_{25}H_{57}O_{26}P_6^-$<br>959.1569<br>959.1586	$C_{30}H_{68}O_{30}P_7^-$<br>1125.1964<br>1125.2004	
c_i^{z-}<br>m/z_{th}<br>m/z_{exp}	*n.e.*	$C_8H_{17}O_8P_2^-$<br>303.0404<br>303.0416	$C_{11}H_{24}O_{12}P_3^-$<br>441.0486<br>441.0497	$C_{14}H_{31}O_{16}P_4^-$<br>579.0568<br>579.0582	$C_{19}H_{42}O_{20}P_5^-$<br>745.0963<br>745.0982	$C_{22}H_{49}O_{24}P_6^-$<br>883.1045<br>883.1046	$C_{25}H_{55}O_{28}P_7^{2-}$<br>510.0527<br>510.0533	$C_{30}H_{66}O_{32}P_8^{2-}$<br>593.0725<br>593.0750	
d_i^{z-}<br>m/z_{th}<br>m/z_{exp}	$C_3H_8O_5P^-$<br>155.0114<br>155.0125	$C_8H_{19}O_9P_2^-$<br>321.0509<br>321.0521	$C_{11}H_{26}O_{13}P_3^-$<br>459.0591<br>459.0605	$C_{14}H_{33}O_{17}P_4^-$<br>597.0674<br>597.0690	$C_{19}H_{44}O_{21}P_5^{1-}$<br>763.1069<br>763.1081	$C_{22}H_{51}O_{25}P_6^-$<br>901.1151<br>901.1161	$C_{25}H_{57}O_{29}P_7^{2-}$<br>519.0580<br>519.0570	$C_{30}H_{68}O_{33}P_8^{2-}$<br>602.0777<br>602.0776	
i →	*1*	*2*	*3*	*4*	*5*	*6*	*7*	*8*	
	**0**	**1**	**0**	**0**	**1**	**0**	**0**	**1**	
	8	*7*	*6*	*5*	*4*	*3*	*2*	*1*	← *j*
	n.e.	*n.d.*	$C_{32}H_{62}O_{28}P_7^{\bullet 2-}$<br>555.5801<br>555.5793	$C_{29}H_{56}O_{24}P_6^{\bullet -}$<br>974.1593<br>974.1603	$C_{26}H_{49}O_{20}P_5^{\bullet -}$<br>836.1511<br>836.1517	$C_{21}H_{38}O_{16}P_4^{\bullet -}$<br>670.1116<br>670.1149	$C_{18}H_{31}O_{12}P_3^{\bullet -}$<br>532.1034<br>532.1050	$C_{15}H_{24}O_8P_2^{\bullet -}$<br>394.0952<br>394.0963	w_i^{z-}<br>m/z_{th}<br>m/z_{exp}
	n.e.	$C_{37}H_{71}O_{31}P_8^{\bullet 2-}$<br>629.5946<br>629.5941	$C_{32}H_{61}O_{27}P_7^{\bullet -}$<br>1094.1569<br>1094.1600	$C_{29}H_{54}O_{23}P_6^{\bullet -}$<br>956.1487<br>956.1505	$C_{26}H_{47}O_{19}P_5^{\bullet -}$<br>818.1405<br>818.1393	$C_{21}H_{36}O_{15}P_4^{\bullet -}$<br>652.1010<br>652.1036	$C_{18}H_{29}O_{11}P_3^{\bullet -}$<br>514.0928<br>514.0941	$C_{15}H_{22}O_7P_2^{\bullet -}$<br>376.0846<br>376.0851	x_i^{z-}<br>m/z_{th}<br>m/z_{exp}
	n.e.	$C_{37}H_{72}O_{29}P_7^{\bullet 2-}$<br>598.6167<br>598.6158	$C_{32}H_{62}O_{25}P_6^{\bullet -}$<br>1032.2011<br>1032.2010	$C_{29}H_{55}O_{21}P_5^{\bullet -}$<br>894.1930<br>894.1931	$C_{26}H_{48}O_{17}P_4^{\bullet -}$<br>756.1848<br>756.1854	$C_{21}H_{37}O_{13}P_3^{\bullet -}$<br>590.1453<br>590.1467	$C_{18}H_{30}O_9P_2^{\bullet -}$<br>452.1371<br>452.1373	$C_{15}H_{23}O_5P^{\bullet -}$<br>314.1289<br>314.1304	y_i^{z-}<br>m/z_{th}<br>m/z_{exp}
	$C_{40}H_{78}O_{32}P_8^{\bullet -}$<br>658.6155<br>658.6137	$C_{37}H_{71}O_{28}P_7^{\bullet -}$<br>1180.2301<br>1180.2320	$C_{32}H_{60}O_{24}P_6^{\bullet -}$<br>1014.1906<br>1014.1917	$C_{29}H_{53}O_{20}P_5^{\bullet -}$<br>876.1824<br>876.1844	$C_{26}H_{46}O_{16}P_4^{\bullet -}$<br>738.1742<br>738.1752	$C_{21}H_{35}O_{12}P_3^{\bullet -}$<br>572.1347<br>572.1367	$C_{18}H_{28}O_8P_2^{\bullet -}$<br>434.1265<br>434.1277	$C_{15}H_{21}O_4P^{\bullet -}$<br>296.1183<br>296.1198	z_i^{z-}<br>m/z_{th}<br>m/z_{exp}

Supplementary Table 3. Accurate mass measurements of sequencing fragments in the CID spectrum recorded for the first block coding for "I" in the "ICR" triblock (Supplementary Fig. 5a), using the $[B1 - 2H]^{2-}$ precursor ion ($C_{40}H_{79}O_{33}P_8^{\bullet 2-}$, *m/z* 667.6208) as an internal standard. 0-bit: $C_3H_7O_4P$; 1-bit: $C_5H_{11}O_4P$. *n.e.*: not expected. *n.d.*: not detected.

a_i^{z-}<br>m/z_{th}<br>m/z_{exp}	$C_{26}H_{43}N_2O_5P^{\bullet -}$<br>494.2915<br>*n.d.*	$C_{31}H_{54}N_2O_9P_2^{\bullet -}$<br>660.3310<br>660.3271	$C_{34}H_{61}N_2O_{13}P_3^{\bullet -}$<br>798.3392<br>798.3386	$C_{37}H_{68}N_2O_{17}P_4^{\bullet -}$<br>936.3474<br>936.3514	$C_{40}H_{75}N_2O_{21}P_5^{\bullet -}$<br>1074.3556<br>1074.3616	$C_{43}H_{82}N_2O_{25}P_6^{\bullet -}$<br>1212.3638<br>1212.3689	$C_{48}H_{93}N_2O_{29}P_7^{\bullet -}$<br>1378.4033<br>1378.3926	$C_{53}H_{103}N_2O_{33}P_8^{\bullet 2-}$<br>771.7178<br>771.7148	
b_i^{z-}<br>m/z_{th}<br>m/z_{exp}	$C_{26}H_{45}N_2O_6P^{\bullet -}$<br>512.3021<br>*n.d.*	$C_{31}H_{56}N_2O_{10}P_2^{\bullet -}$<br>678.3416<br>678.3445	$C_{34}H_{63}N_2O_{14}P_3^{\bullet -}$<br>816.3498<br>816.3535	$C_{37}H_{70}N_2O_{18}P_4^{\bullet -}$<br>954.3580<br>954.3578	$C_{40}H_{77}N_2O_{22}P_5^{\bullet -}$<br>1092.3662<br>1092.3698	$C_{43}H_{84}N_2O_{26}P_6^{\bullet -}$<br>1230.3744<br>1230.3751	$C_{48}H_{94}N_2O_{30}P_7^{\bullet 2-}$<br>697.7033<br>*n.d.*	$C_{53}H_{105}N_2O_{34}P_8^{\bullet 2-}$<br>780.7230<br>*n.d.*	
c_i^{z-}<br>m/z_{th}<br>m/z_{exp}	$C_{26}H_{44}N_2O_8P_2^{\bullet -}$<br>574.2578<br>574.2584	$C_{31}H_{55}N_2O_{12}P_3^{\bullet -}$<br>740.2973<br>740.2969	$C_{34}H_{62}N_2O_{16}P_4^{\bullet -}$<br>878.3055<br>878.2979	$C_{37}H_{69}N_2O_{20}P_5^{\bullet -}$<br>1016.3137<br>1016.3192	$C_{40}H_{76}N_2O_{24}P_6^{\bullet -}$<br>1154.3219<br>1154.3201	$C_{43}H_{83}N_2O_{28}P_7^{\bullet -}$<br>1292.3301<br>1292.3383	$C_{48}H_{93}N_2O_{32}P_8^{\bullet 2-}$<br>728.6812<br>728.6801	$C_{53}H_{104}N_2O_{36}P_9^{\bullet 2-}$<br>811.7009<br>811.6976	
d_i^{z-}<br>m/z_{th}<br>m/z_{exp}	$C_{26}H_{46}N_2O_9P_2^{\bullet -}$<br>592.2684<br>592.2700	$C_{31}H_{57}N_2O_{13}P_3^{\bullet -}$<br>758.3079<br>758.3085	$C_{34}H_{64}N_2O_{17}P_4^{\bullet -}$<br>896.3161<br>896.3140	$C_{37}H_{71}N_2O_{21}P_5^{\bullet -}$<br>1034.3244<br>1034.3289	$C_{40}H_{78}N_2O_{25}P_6^{\bullet -}$<br>1172.3325<br>1172.3370	$C_{43}H_{84}N_2O_{29}P_7^{\bullet 2-}$<br>654.6667<br>654.6644	$C_{48}H_{95}N_2O_{33}P_8^{\bullet 2-}$<br>737.6865<br>737.6872	$C_{53}H_{106}N_2O_{37}P_9^{\bullet 2-}$<br>820.7062<br>820.7112	
$i \rightarrow$	*1*	*2*	*3*	*4*	*5*	*6*	*7*	*8*	
	**0**	**1**	**0**	**0**	**0**	**0**	**1**	**1**	
	8	*7*	*6*	*5*	*4*	*3*	*2*	*1*	$\leftarrow j$
	$C_{40}H_{80}O_{36}P_9^{\bullet 2-}$<br>707.6039<br>707.6061	$C_{37}H_{73}O_{32}P_8^{\bullet 2-}$<br>638.5999<br>638.6000	$C_{32}H_{62}O_{28}P_7^{\bullet 2-}$<br>555.5801<br>555.5816	$C_{29}H_{56}O_{24}P_6^{\bullet -}$<br>974.1593<br>974.1625	$C_{26}H_{49}O_{20}P_5^{\bullet -}$<br>836.1511<br>836.1526	$C_{23}H_{42}O_{16}P_4^{\bullet -}$<br>698.1429<br>698.1437	$C_{20}H_{35}O_{12}P_3^{\bullet -}$<br>560.1347<br>560.1353	$C_{15}H_{24}O_8P_2^{\bullet -}$<br>394.0952<br>394.0945	w_i^{z-}<br>m/z_{th}<br>m/z_{exp}
	$C_{40}H_{78}O_{35}P_9^{\bullet 2-}$<br>698.5987<br>698.5981	$C_{37}H_{71}O_{31}P_8^{\bullet 2-}$<br>629.5946<br>629.5928	$C_{32}H_{60}O_{27}P_7^{\bullet 2-}$<br>546.5748<br>546.5737	$C_{29}H_{54}O_{23}P_6^{\bullet -}$<br>956.1487<br>956.1475	$C_{26}H_{47}O_{19}P_5^{\bullet -}$<br>818.1405<br>818.1431	$C_{23}H_{40}O_{15}P_4^{\bullet -}$<br>680.1323<br>680.1331	$C_{20}H_{33}O_{11}P_3^{\bullet -}$<br>542.1241<br>542.1228	$C_{15}H_{22}O_7P_2^{\bullet -}$<br>376.0846<br>376.0852	x_i^{z-}<br>m/z_{th}<br>m/z_{exp}
	$C_{40}H_{79}O_{33}P_8^{\bullet 2-}$<br>667.6208<br>667.6218	$C_{37}H_{73}O_{29}P_7^{\bullet -}$<br>1198.2406<br>1198.2409	$C_{32}H_{62}O_{25}P_6^{\bullet -}$<br>1032.2011<br>1032.1963	$C_{29}H_{55}O_{21}P_5^{\bullet -}$<br>894.1930<br>894.1979	$C_{26}H_{48}O_{17}P_4^{\bullet -}$<br>756.1848<br>756.1830	$C_{23}H_{41}O_{13}P_3^{\bullet -}$<br>618.1766<br>618.1768	$C_{20}H_{34}O_9P_2^{\bullet -}$<br>480.1684<br>480.1705	$C_{15}H_{23}O_5P^{\bullet -}$<br>314.1289<br>*n.d.*	y_i^{z-}<br>m/z_{th}<br>m/z_{exp}
	$C_{40}H_{78}O_{32}P_8^{\bullet -}$<br>1318.2383<br>1318.2371	$C_{37}H_{71}O_{28}P_7^{\bullet -}$<br>1180.2301<br>1180.2319	$C_{32}H_{60}O_{24}P_6^{\bullet -}$<br>1014.1906<br>1014.1899	$C_{29}H_{53}O_{20}P_5^{\bullet -}$<br>876.1824<br>876.1837	$C_{26}H_{46}O_{16}P_4^{\bullet -}$<br>738.1742<br>738.1761	$C_{23}H_{39}O_{12}P_3^{\bullet -}$<br>600.1660<br>600.1682	$C_{20}H_{32}O_8P_2^{\bullet -}$<br>462.1578<br>462.1600	$C_{15}H_{21}O_4P^{\bullet -}$<br>296.1183<br>*n.d.*	z_i^{z-}<br>m/z_{th}<br>m/z_{exp}

Supplementary Table 4. Accurate mass measurements of sequencing fragments in the CID spectrum recorded for the second block coding for "C" in the "ICR" triblock (Supplementary Fig. 5b), using the $[B2 - 2H]^{2-}$ precursor ion ($C_{63}H_{117}N_2O_{37}P_9^{\bullet\bullet 2-}$, *m/z* 886.2492) as an internal standard. 0-bit: $C_3H_7O_4P$; 1-bit: $C_5H_{11}O_4P$. *n.e.*: not expected. *n.d.*: not detected.

a_i^{z-}	$C_{26}H_{37}N_2O_5P^{\bullet-}$	$C_{31}H_{48}N_2O_9P_2^{\bullet-}$	$C_{34}H_{55}N_2O_{13}P_3^{\bullet-}$	$C_{39}H_{66}N_2O_{17}P_4^{\bullet-}$	$C_{42}H_{73}N_2O_{21}P_5^{\bullet-}$	$C_{45}H_{80}N_2O_{25}P_6^{\bullet-}$	$C_{50}H_{90}N_2O_{29}P_7^{\bullet 2-}$	$C_{53}H_{97}N_2O_{33}P_8^{\bullet 2-}$	
m/z_{th}	488.2446	654.2841	792.2923	958.3318	1096.3399	1234.3481	699.6902	768.6943	
m/z_{exp}	488.2451	654.2851	792.2936	958.3338	1096.3413	1234.3500	699.6932	768.6998	
b_i^{z-}	$C_{26}H_{39}N_2O_6P^{\bullet-}$	$C_{31}H_{50}N_2O_{10}P_2^{\bullet-}$	$C_{34}H_{57}N_2O_{14}P_3^{\bullet-}$	$C_{39}H_{68}N_2O_{18}P_4^{\bullet-}$	$C_{42}H_{75}N_2O_{22}P_5^{\bullet-}$	$C_{45}H_{82}N_2O_{26}P_6^{\bullet-}$	$C_{50}H_{92}N_2O_{30}P_7^{\bullet 2-}$		
m/z_{th}	506.2551	672.2946	810.3028	976.3423	1114.3505	1252.3587	708.6955	*n.e.*	
m/z_{exp}	506.2568	672.2958	810.3027	976.3444	1114.3544	1252.3617	708.6953		
c_i^{z-}	$C_{26}H_{38}N_2O_8P_2^{\bullet-}$	$C_{31}H_{49}N_2O_{12}P_3^{\bullet-}$	$C_{34}H_{56}N_2O_{16}P_4^{\bullet-}$	$C_{39}H_{67}N_2O_{20}P_5^{\bullet-}$	$C_{42}H_{74}N_2O_{24}P_6^{\bullet-}$	$C_{45}H_{81}N_2O_{28}P_7^{\bullet-}$	$C_{50}H_{91}N_2O_{32}P_8^{\bullet 2-}$		
m/z_{th}	568.2109	734.2504	872.2586	1038.2981	1176.3063	1314.3145	739.6733	*n.e.*	
m/z_{exp}	568.2115	734.2498	872.2578	1038.2990	1176.3074	1314.3130	739.6716		
d_i^{z-}	$C_{26}H_{40}N_2O_9P_2^{\bullet-}$	$C_{31}H_{51}N_2O_{13}P_3^{\bullet-}$	$C_{34}H_{58}N_2O_{17}P_4^{\bullet-}$	$C_{39}H_{69}N_2O_{21}P_5^{\bullet-}$	$C_{42}H_{76}N_2O_{25}P_6^{\bullet-}$	$C_{45}H_{82}N_2O_{29}P_7^{\bullet 2-}$	$C_{50}H_{93}N_2O_{33}P_8^{\bullet 2-}$		
m/z_{th}	586.2215	752.2610	890.2692	1056.3086	1194.3168	665.6589	748.6786	*n.e.*	
m/z_{exp}	586.2244	752.2611	890.2686	1056.3102	1194.3182	665.6599	*n.d.*		
$i \rightarrow$	*1*	*2*	*3*	*4*	*5*	*6*	*7*	*8*	
	**0**	**1**	**0**	**1**	**0**	**0**	**1**	**0**	
	8	*7*	*6*	*5*	*4*	*3*	*2*	*1*	$\leftarrow j$
	$C_{30}H_{68}O_{33}P_8^{2-}$	$C_{27}H_{61}O_{29}P_7^{2-}$	$C_{22}H_{51}O_{25}P_6^{-}$	$C_{19}H_{44}O_{21}P_5^{-}$	$C_{14}H_{33}O_{17}P_4^{-}$	$C_{11}H_{26}O_{13}P_3^{-}$	$C_8H_{19}O_9P_2^{-}$	$C_3H_8O_5P^{-}$	w_i^{z-}
	602.0777	533.0736	901.1151	763.1069	597.0674	459.0592	321.0510	155.0115	m/z_{th}
	602.0804	533.0744	901.1161	763.1087	597.0667	459.0600	321.0520	155.0135	m/z_{exp}
	$C_{30}H_{66}O_{32}P_8^{2-}$	$C_{27}H_{59}O_{28}P_7^{2-}$	$C_{22}H_{49}O_{24}P_6^{-}$	$C_{19}H_{42}O_{20}P_5^{-}$	$C_{14}H_{31}O_{16}P_4^{-}$	$C_{11}H_{24}O_{12}P_3^{-}$	$C_8H_{17}O_8P_2^{-}$		x_i^{z-}
	593.0725	524.0684	883.1045	745.0963	579.0568	441.0486	303.0404	*n.e.*	m/z_{th}
	593.0730	524.0692	883.1050	745.0989	579.0592	441.0502	303.0417		m/z_{exp}
	$C_{30}H_{67}O_{30}P_7^{2-}$	$C_{27}H_{61}O_{26}P_6^{-}$	$C_{22}H_{50}O_{22}P_5^{-}$	$C_{19}H_{43}O_{18}P_4^{-}$	$C_{14}H_{32}O_{14}P_3^{-}$	$C_{11}H_{25}O_{10}P_2^{-}$	$C_8H_{18}O_6P^{-}$		y_i^{z-}
	562.0946	987.1882	821.1487	683.1405	517.1010	379.0928	241.0847	*n.e.*	m/z_{th}
	562.0955	987.1882	821.1545	683.1407	517.1034	379.0940	241.0859		m/z_{exp}
	$C_{30}H_{65}O_{29}P_7^{2-}$	$C_{27}H_{59}O_{25}P_6^{-}$	$C_{22}H_{48}O_{21}P_5^{-}$	$C_{19}H_{41}O_{17}P_4^{-}$	$C_{14}H_{30}O_{13}P_3^{-}$	$C_{11}H_{23}O_9P_2^{-}$	$C_8H_{16}O_5P^{-}$		z_i^{z-}
	553.0893	969.1777	803.1382	665.1300	499.0905	361.0823	223.0741	*n.e.*	m/z_{th}
	553.0901	969.1796	803.1393	665.1309	499.0924	361.0832	223.0791		m/z_{exp}

Supplementary Table 5. Accurate mass measurements of sequencing fragments in the CID spectrum recorded for the last block coding for “R” in the “ICR” triblock (Supplementary Fig. 5c), using the $[B3 - 2H]^{2-}$ precursor ion ($C_{53}H_{99}N_2O_{34}P_8^{\bullet 2-}$, *m/z* 777.6996) as an internal standard. 0-bit: $C_3H_7O_4P$; 1-bit: $C_5H_{11}O_4P$. *n.e.*: not expected. *n.d.*: not detected.

A	01000001	H	01001000	O	01001111	V	01010110
a	01100001	h	01101000	o	01101111	v	01110110
B	01000010	I	01001001	P	01010000	W	01010111
b	01100010	i	01101001	p	01110000	w	01110111
C	01000011	J	01001010	Q	01010001	X	01011000
c	01100011	j	01101010	q	01110001	x	01111000
D	01000100	K	01001011	R	01010010	Y	01011001
d	01100100	k	01101011	r	01110010	y	01111001
E	01000101	L	01001100	S	01010011	Z	01011010
e	01100101	l	01101100	s	01110011	z	01111010
F	01000110	M	01001101	T	01010100	.	00101110
f	01100110	m	01101101	t	01110100	;	00111011
G	01000111	N	01001110	U	01010101	?	00111111
g	01100111	n	01101110	u	01110101	!	00100001

Supplementary Table 6. ASCII code for uppercase and lowercase letters of the Latin alphabet, colored as a function of their 0_i1_j co-monomeric composition, with i/j: 6/2 (blue), 5/3 (yellow), 4/4 (green), 3/5 (orange), 2/6 (pink). Similar data are also shown for a few punctuation marks in the lower right side of the table.

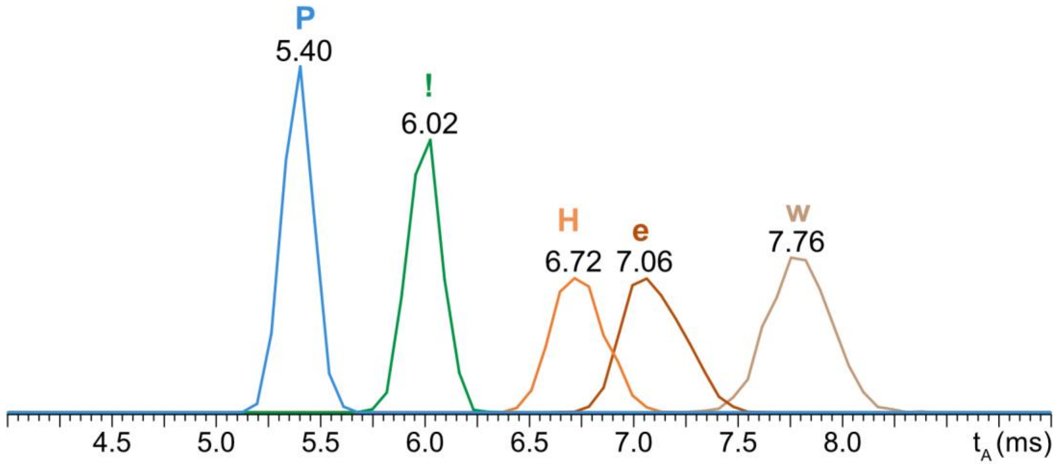

Supplementary Fig. 6. IMS traces extracted for $[B1 - 2H]^{2-}$ at *m/z* 653.6 (in blue), $[B5 - 2H]^{2-}$ at *m/z* 738.7 (in green), $[B2 - 2H]^{2-}$ at *m/z* 844.2 (orange), $[B3 - 2H]^{2-}$ at *m/z* 872.2 (dark brown) and $[B4 - 2H]^{2-}$ at *m/z* 928.3 (pale brown) after performing MS-(CID)-IMS-MS of the PPDE pentablock coding for “PHew!”.

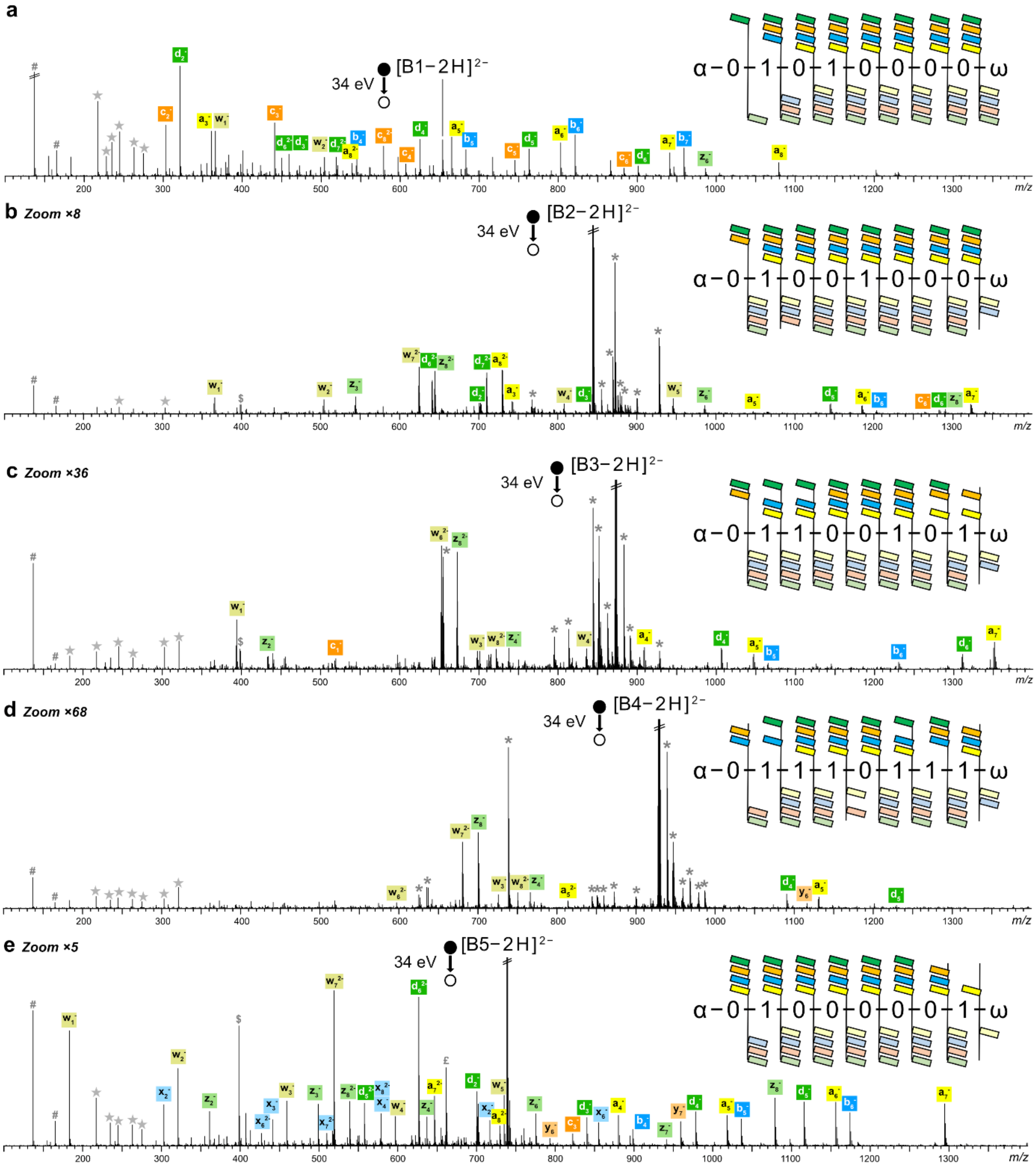

Supplementary Fig. 7. Reading "PHew!" coded in a PPDE pentablock. Sequencing of the five blocks of the "PHew!" b-PPDE from CID spectra recorded for **a** $[B1 - 2H]^{2-}$ at *m/z* 653.6, **b** $[B2 - 2H]^{2-}$ at *m/z* 844.2, **c** $[B3 - 2H]^{2-}$ at *m/z* 872.2, **d** $[B4 - 2H]^{2-}$ at *m/z* 928.3 and **e** $[B5 - 2H]^{2-}$ at *m/z* 738.7, with inset schemes showing full sequence coverage. Accurate mass measurements of sequencing fragments are reported in Supplementary Tables 7-11. Grey symbols: $: tag; #: deprotonated monomer; £: loss of TEMPO; stars: internal fragments.

a_i^{z-}<br>m/z_{th}<br>m/z_{exp}	*n.e.*	*n.e.*	$C_{11}H_{23}O_9P_2^-$<br>361.0823<br>361.0833	$C_{16}H_{34}O_{13}P_3^-$<br>527.1218<br>527.1238	$C_{19}H_{41}O_{17}P_4^-$<br>665.1300<br>665.1317	$C_{22}H_{48}O_{21}P_5^-$<br>803.1382<br>803.1401	$C_{25}H_{55}O_{25}P_6^-$<br>941.1464<br>941.1474	$C_{28}H_{62}O_{29}P_7^-$<br>1079.1546<br>1079.1572	
b_i^{z-}<br>m/z_{th}<br>m/z_{exp}	*n.e.*	$C_8H_{18}O_6P^-$<br>241.0847<br>241.0856	$C_{11}H_{25}O_{10}P_2^-$<br>379.0928<br>379.0939	$C_{16}H_{36}O_{14}P_3^-$<br>545.1323<br>545.1311	$C_{19}H_{43}O_{18}P_4^-$<br>683.1405<br>683.1414	$C_{22}H_{50}O_{22}P_5^-$<br>821.1487<br>821.1483	$C_{25}H_{57}O_{26}P_6^-$<br>959.1569<br>959.1586	$C_{28}H_{64}O_{30}P_7^-$<br>1097.1651<br>1097.1675	
c_i^{z-}<br>m/z_{th}<br>m/z_{exp}	*n.e.*	$C_8H_{17}O_8P_2^-$<br>303.0404<br>303.0415	$C_{11}H_{24}O_{12}P_3^-$<br>441.0486<br>441.0499	$C_{16}H_{35}O_{16}P_4^-$<br>607.0881<br>607.0898	$C_{19}H_{42}O_{20}P_5^-$<br>745.0963<br>745.1005	$C_{22}H_{49}O_{24}P_6^-$<br>883.1045<br>883.1071	$C_{25}H_{55}O_{28}P_7^{2-}$<br>510.0527<br>510.0569	$C_{28}H_{62}O_{32}P_8^{2-}$<br>579.0568<br>579.0577	
d_i^{z-}<br>m/z_{th}<br>m/z_{exp}	$C_3H_8O_5P^-$<br>155.0114<br>155.0124	$C_8H_{19}O_9P_2^-$<br>321.0509<br>321.0523	$C_{11}H_{26}O_{13}P_3^-$<br>459.0591<br>459.0602	$C_{16}H_{37}O_{17}P_4^-$<br>625.0987<br>625.1000	$C_{19}H_{44}O_{21}P_5^{1-}$<br>763.1069<br>763.1069	$C_{22}H_{51}O_{25}P_6^-$<br>901.1151<br>901.1174	$C_{25}H_{57}O_{29}P_7^{2-}$<br>519.0580<br>519.0585	$C_{28}H_{64}O_{33}P_8^{2-}$<br>588.0621<br>588.0640	
$i \rightarrow$	*1*	*2*	*3*	*4*	*5*	*6*	*7*	*8*	
	**0**	**1**	**0**	**1**	**0**	**0**	**0**	**0**	
	8	*7*	*6*	*5*	*4*	*3*	*2*	*1*	$\leftarrow j$
	n.e.	*n.d.*	$C_{30}H_{58}O_{28}P_7^{\bullet 2-}$<br>541.5645	$C_{27}H_{52}O_{24}P_6^{\bullet -}$<br>946.1280<br>946.1279	$C_{22}H_{41}O_{20}P_5^{\bullet -}$<br>780.0885<br>780.0859	$C_{19}H_{34}O_{16}P_4^{\bullet -}$<br>642.0803<br>642.0824	$C_{16}H_{27}O_{12}P_3^{\bullet -}$<br>504.0721<br>504.0741	$C_{13}H_{20}O_8P_2^{\bullet -}$<br>366.0639<br>366.0657	w_i^{z-}<br>m/z_{th}<br>m/z_{exp}
	n.e.	$C_{35}H_{68}O_{31}P_8^{\bullet -}$<br>1232.1651<br>*n.d.*	$C_{30}H_{57}O_{27}P_7^{\bullet -}$<br>1066.1256<br>1066.1320	$C_{27}H_{50}O_{23}P_6^{\bullet -}$<br>928.1174<br>928.1196	$C_{22}H_{39}O_{19}P_5^{\bullet -}$<br>762.0779<br>762.0746	$C_{19}H_{32}O_{15}P_4^{\bullet -}$<br>624.0697<br>624.0718	$C_{16}H_{25}O_{11}P_3^{\bullet -}$<br>486.0615<br>486.0640	$C_{13}H_{18}O_7P_2^{\bullet -}$<br>348.0533<br>348.0549	x_i^{z-}<br>m/z_{th}<br>m/z_{exp}
	n.e.	$C_{35}H_{68}O_{29}P_7^{\bullet 2-}$<br>584.6010<br>584.6059	$C_{30}H_{58}O_{25}P_6^{\bullet -}$<br>1004.1698<br>1004.1742	$C_{27}H_{51}O_{21}P_5^{\bullet -}$<br>866.1616<br>866.1649	$C_{22}H_{40}O_{17}P_4^{\bullet -}$<br>700.1222<br>700.1249	$C_{19}H_{33}O_{13}P_3^{\bullet -}$<br>562.1140<br>562.1172	$C_{16}H_{26}O_9P_2^{\bullet -}$<br>424.1058<br>424.1094	$C_{13}H_{19}O_5P^{\bullet -}$<br>286.0976<br>286.1009	y_i^{z-}<br>m/z_{th}<br>m/z_{exp}
	$C_{38}H_{73}O_{32}P_8^{\bullet 2-}$<br>644.5999<br>644.6042	$C_{35}H_{66}O_{28}P_7^{\bullet 2-}$<br>575.5958<br>575.5968	$C_{30}H_{55}O_{24}P_6^{\bullet 2-}$<br>492.5760<br>492.5735	$C_{27}H_{49}O_{20}P_5^{\bullet -}$<br>848.1511<br>848.1470	$C_{22}H_{38}O_{16}P_4^{\bullet -}$<br>682.1116<br>682.1140	$C_{19}H_{31}O_{12}P_3^{\bullet -}$<br>544.1034<br>544.1055	$C_{16}H_{24}O_8P_2^{\bullet -}$<br>406.0952<br>406.0967	$C_{13}H_{17}O_4P^{\bullet -}$<br>268.0870<br>268.0862	z_i^{z-}<br>m/z_{th}<br>m/z_{exp}

Supplementary Table 7. Accurate mass measurements of sequencing fragments in the CID spectrum recorded for the first block coding for "P" in the "PHew!" pentablock (Supplementary Fig. 7a), using the $[B1 - 2H]^{2-}$ precursor ion ($C_{38}H_{77}O_{33}P_8^{\bullet 2-}$, *m/z* 653.6051) as an internal standard. 0-bit: $C_3H_7O_4P$; 1-bit: $C_5H_{11}O_4P$. *n.e.*: not expected. *n.d.*: not detected.

a_i^{z-}	$C_{22}H_{35}N_2O_5P^{\bullet-}$	$C_{27}H_{46}N_2O_9P_2^{\bullet-}$	$C_{30}H_{53}N_2O_{13}P_3^{\bullet-}$	$C_{33}H_{60}N_2O_{17}P_4^{\bullet-}$	$C_{38}H_{71}N_2O_{21}P_5^{\bullet-}$	$C_{41}H_{78}N_2O_{25}P_6^{\bullet-}$	$C_{44}H_{85}N_2O_{29}P_7^{\bullet-}$	$C_{47}H_{91}N_2O_{33}P_8^{\bullet 2-}$	
m/z_{th}	438.2289	604.2684	742.2766	880.2848	1046.3243	1184.3325	1322.3407	729.6708	
m/z_{exp}	*n.d.*	604.2737	742.2756	880.2891	1046.3296	1184.3282	1322.3424	729.6696	
b_i^{z-}	$C_{22}H_{37}N_2O_6P^{\bullet-}$	$C_{27}H_{48}N_2O_{10}P_2^{\bullet-}$	$C_{30}H_{55}N_2O_{14}P_3^{\bullet-}$	$C_{33}H_{62}N_2O_{18}P_4^{\bullet-}$	$C_{38}H_{73}N_2O_{22}P_5^{\bullet-}$	$C_{41}H_{80}N_2O_{26}P_6^{\bullet-}$	$C_{44}H_{86}N_2O_{30}P_7^{\bullet 2-}$	$C_{47}H_{93}N_2O_{34}P_8^{\bullet 2-}$	
m/z_{th}	456.2395	622.2790	760.2872	898.2954	1064.3349	1202.3431	669.6720	738.6761	
m/z_{exp}	*n.d.*	622.2831	760.2930	898.2928	1064.3391	1202.3422	669.6728	738.6792	
c_i^{z-}	$C_{22}H_{36}N_2O_8P_2^{\bullet-}$	$C_{27}H_{47}N_2O_{12}P_3^{\bullet-}$	$C_{30}H_{54}N_2O_{16}P_4^{\bullet-}$	$C_{33}H_{61}N_2O_{20}P_5^{\bullet-}$	$C_{38}H_{72}N_2O_{24}P_6^{\bullet-}$	$C_{41}H_{79}N_2O_{28}P_7^{\bullet-}$	$C_{44}H_{85}N_2O_{32}P_8^{\bullet 2-}$	$C_{47}H_{92}N_2O_{36}P_9^{\bullet 2-}$	
m/z_{th}	518.1952	684.2347	822.2429	960.2511	1126.2906	1264.2988	700.6499	769.6540	
m/z_{exp}	536.2108	684.2319	822.2393	960.2485	1126.2864	1264.2986	700.6553	769.6571	
d_i^{z-}	$C_{22}H_{38}N_2O_9P_2^{\bullet-}$	$C_{27}H_{49}N_2O_{13}P_3^{\bullet-}$	$C_{30}H_{56}N_2O_{17}P_4^{\bullet-}$	$C_{33}H_{63}N_2O_{21}P_5^{\bullet-}$	$C_{38}H_{74}N_2O_{25}P_6^{\bullet-}$	$C_{41}H_{80}N_2O_{29}P_7^{\bullet 2-}$	$C_{44}H_{87}N_2O_{33}P_8^{\bullet 2-}$	$C_{47}H_{94}N_2O_{37}P_9^{\bullet 2-}$	
m/z_{th}	536.2058	702.2453	840.2535	978.2617	1144.3012	640.6511	709.6552	778.6593	
m/z_{exp}	536.2108	702.2476	840.2509	978.2626	1144.3051	640.6558	709.6555	778.6598	
$i \rightarrow$	*1*	*2*	*3*	*4*	*5*	*6*	*7*	*8*	
	**0**	**1**	**0**	**0**	**1**	**0**	**0**	**0**	
	8	*7*	*6*	*5*	*4*	*3*	*2*	*1*	$\leftarrow j$
	$C_{38}H_{76}O_{36}P_9^{\bullet 2-}$	$C_{35}H_{69}O_{32}P_8^{\bullet 2-}$	$C_{30}H_{58}O_{28}P_7^{\bullet 2-}$	$C_{27}H_{51}O_{24}P_6^{\bullet 2-}$	$C_{24}H_{45}O_{20}P_5^{\bullet-}$	$C_{19}H_{34}O_{16}P_4^{\bullet-}$	$C_{16}H_{27}O_{12}P_3^{\bullet-}$	$C_{13}H_{20}O_8P_2^{\bullet-}$	w_i^{z-}
	693.5883	624.5842	541.5645	472.5604	808.1198	642.0803	504.0721	366.0639	m/z_{th}
	693.5860	624.5858	541.5656	472.5602	808.1205	642.0839	504.0738	366.0641	m/z_{exp}
	$C_{38}H_{74}O_{35}P_9^{\bullet 2-}$	$C_{35}H_{67}O_{31}P_8^{\bullet 2-}$	$C_{30}H_{56}O_{27}P_7^{\bullet 2-}$	$C_{27}H_{50}O_{23}P_6^{\bullet-}$	$C_{24}H_{43}O_{19}P_5^{\bullet-}$	$C_{19}H_{32}O_{15}P_4^{\bullet-}$	$C_{16}H_{25}O_{11}P_3^{\bullet-}$	$C_{13}H_{18}O_7P_2^{\bullet-}$	x_i^{z-}
	684.5830	615.5789	532.5592	928.1174	790.1092	624.0697	486.0615	348.0533	m/z_{th}
	684.5836	615.5745	532.5632	928.1177	790.1132	624.0702	486.0696	348.0560	m/z_{exp}
	$C_{38}H_{75}O_{33}P_8^{\bullet 2-}$	$C_{35}H_{69}O_{29}P_7^{\bullet-}$	$C_{30}H_{58}O_{25}P_6^{\bullet-}$	$C_{27}H_{51}O_{21}P_5^{\bullet-}$	$C_{24}H_{44}O_{17}P_4^{\bullet-}$	$C_{19}H_{33}O_{13}P_3^{\bullet-}$	$C_{16}H_{26}O_9P_2^{\bullet-}$	$C_{13}H_{19}O_5P^{\bullet-}$	y_i^{z-}
	653.6051	1170.2093	1004.1698	866.1616	728.1535	562.1140	424.1058	286.0976	m/z_{th}
	653.6066	1170.2147	1004.1642	866.1669	728.1537	562.1127	424.1093	*n.d.*	m/z_{exp}
	$C_{38}H_{73}O_{32}P_8^{\bullet 2-}$	$C_{35}H_{67}O_{28}P_7^{\bullet-}$	$C_{30}H_{56}O_{24}P_6^{\bullet-}$	$C_{27}H_{49}O_{20}P_5^{\bullet-}$	$C_{24}H_{42}O_{16}P_4^{\bullet-}$	$C_{19}H_{31}O_{12}P_3^{\bullet-}$	$C_{16}H_{24}O_8P_2^{\bullet-}$	$C_{13}H_{17}O_4P^{\bullet-}$	z_i^{z-}
	644.5999	1152.1988	986.1593	848.1511	710.1429	544.1034	406.0952	268.0870	m/z_{th}
	644.5997	*n.d.*	986.1617	848.1507	710.1496	544.1046	406.1008	*n.d.*	m/z_{exp}

Supplementary Table 8. Accurate mass measurements of sequencing fragments in the CID spectrum recorded for the second block coding for "H" in the "PHew!" pentablock (Supplementary Fig. 7b), using the $[B2 - 2H]^{2-}$ precursor ion ($C_{57}H_{105}N_2O_{37}P_9^{\bullet\bullet 2-}$, *m/z* 844.2023) as an internal standard. 0-bit: $C_3H_7O_4P$; 1-bit: $C_5H_{11}O_4P$. *n.e.*: not expected. *n.d.*: not detected.

a_i^{z-}<br>m/z_{th}<br>m/z_{exp}	$C_{22}H_{35}N_2O_5P^{\bullet-}$<br>438.2289<br>*n.d.*	$C_{27}H_{46}N_2O_9P_2^{\bullet-}$<br>604.2684<br>604.2712	$C_{32}H_{57}N_2O_{13}P_3^{\bullet-}$<br>770.3079<br>770.3016	$C_{35}H_{64}N_2O_{17}P_4^{\bullet-}$<br>908.3161<br>908.3203	$C_{38}H_{71}N_2O_{21}P_5^{\bullet-}$<br>1046.3243<br>1046.3282	$C_{43}H_{82}N_2O_{25}P_6^{\bullet-}$<br>1212.3638<br>1212.3657	$C_{46}H_{89}N_2O_{29}P_7^{\bullet-}$<br>1350.3720<br>1350.3741	$C_{51}H_{99}N_2O_{33}P_8^{\bullet 2-}$<br>757.7021<br>757.7036	
b_i^{z-}<br>m/z_{th}<br>m/z_{exp}	$C_{22}H_{37}N_2O_6P^{\bullet-}$<br>456.2395<br>*n.d.*	$C_{27}H_{48}N_2O_{10}P_2^{\bullet-}$<br>622.2790<br>622.2792	$C_{32}H_{59}N_2O_{14}P_3^{\bullet-}$<br>788.3185<br>788.3165	$C_{35}H_{66}N_2O_{18}P_4^{\bullet-}$<br>926.3267<br>926.3282	$C_{38}H_{73}N_2O_{22}P_5^{\bullet-}$<br>1064.3349<br>1064.3338	$C_{43}H_{84}N_2O_{26}P_6^{\bullet-}$<br>1230.3744<br>1230.3751	$C_{46}H_{90}N_2O_{30}P_7^{\bullet 2-}$<br>683.6876<br>*n.d.*	$C_{51}H_{101}N_2O_{34}P_8^{\bullet 2-}$<br>766.7074<br>*n.d.*	
c_i^{z-}<br>m/z_{th}<br>m/z_{exp}	$C_{22}H_{36}N_2O_8P_2^{\bullet-}$<br>518.1952<br>518.2004	$C_{27}H_{47}N_2O_{12}P_3^{\bullet-}$<br>684.2347<br>*n.d.*	$C_{32}H_{58}N_2O_{16}P_4^{\bullet-}$<br>850.2742<br>*n.d.*	$C_{35}H_{65}N_2O_{20}P_5^{\bullet-}$<br>988.2824<br>988.2822	$C_{38}H_{72}N_2O_{24}P_6^{\bullet-}$<br>1126.2906<br>1126.2927	$C_{43}H_{83}N_2O_{28}P_7^{\bullet-}$<br>1292.3301<br>1292.3353	$C_{46}H_{89}N_2O_{32}P_8^{\bullet 2-}$<br>714.6655<br>714.6661	$C_{51}H_{100}N_2O_{36}P_9^{\bullet 2-}$<br>797.6853<br>797.6887	
d_i^{z-}<br>m/z_{th}<br>m/z_{exp}	$C_{22}H_{38}N_2O_9P_2^{\bullet-}$<br>536.2058<br>536.2065	$C_{27}H_{49}N_2O_{13}P_3^{\bullet-}$<br>702.2453<br>702.2492	$C_{32}H_{60}N_2O_{17}P_4^{\bullet-}$<br>868.2848<br>868.2888	$C_{35}H_{67}N_2O_{21}P_5^{\bullet-}$<br>1006.2930<br>1006.2892	$C_{38}H_{74}N_2O_{25}P_6^{\bullet-}$<br>1144.3012<br>1144.3057	$C_{43}H_{84}N_2O_{29}P_7^{\bullet 2-}$<br>654.6667<br>654.6674	$C_{46}H_{91}N_2O_{33}P_8^{\bullet 2-}$<br>723.6708<br>723.6721	$C_{51}H_{102}N_2O_{37}P_9^{\bullet 2-}$<br>806.6906<br>*n.d.*	
i →	*1*	*2*	*3*	*4*	*5*	*6*	*7*	*8*	
	**0**	**1**	**1**	**0**	**0**	**1**	**0**	**1**	
	8	*7*	*6*	*5*	*4*	*3*	*2*	*1*	← *j*
	$C_{42}H_{84}O_{36}P_9^{\bullet 2-}$<br>721.6196<br>721.6168	$C_{39}H_{77}O_{32}P_8^{\bullet 2-}$<br>652.6155<br>652.6150	$C_{34}H_{66}O_{28}P_7^{\bullet 2-}$<br>569.5958<br>569.5916	$C_{29}H_{56}O_{24}P_6^{\bullet-}$<br>974.1593<br>974.1533	$C_{26}H_{49}O_{20}P_5^{\bullet-}$<br>836.1511<br>836.1526	$C_{23}H_{42}O_{16}P_4^{\bullet-}$<br>698.1429<br>698.1453	$C_{18}H_{31}O_{12}P_3^{\bullet-}$<br>532.1034<br>532.1055	$C_{15}H_{24}O_8P_2^{\bullet-}$<br>394.0952<br>394.0955	w_i^{z-}<br>m/z_{th}<br>m/z_{exp}
	$C_{42}H_{82}O_{35}P_9^{\bullet 2-}$<br>712.6143<br>712.6160	$C_{39}H_{75}O_{31}P_8^{\bullet 2-}$<br>643.6102<br>643.6147	$C_{34}H_{65}O_{27}P_7^{\bullet-}$<br>1122.1882<br>1122.1889	$C_{29}H_{54}O_{23}P_6^{\bullet-}$<br>956.1487<br>956.1447	$C_{26}H_{47}O_{19}P_5^{\bullet-}$<br>818.1405<br>818.1487	$C_{23}H_{40}O_{15}P_4^{\bullet-}$<br>680.1323<br>680.1294	$C_{18}H_{29}O_{11}P_3^{\bullet-}$<br>514.0928<br>514.0948	$C_{15}H_{22}O_7P_2^{\bullet-}$<br>376.0846<br>376.0850	x_i^{z-}<br>m/z_{th}<br>m/z_{exp}
	$C_{42}H_{83}O_{33}P_8^{\bullet 2-}$<br>681.6364<br>681.6325	$C_{39}H_{77}O_{29}P_7^{\bullet-}$<br>1226.2719<br>1226.2673	$C_{34}H_{66}O_{25}P_6^{\bullet-}$<br>1060.2324<br>1060.2347	$C_{29}H_{55}O_{21}P_5^{\bullet-}$<br>894.1930<br>894.1994	$C_{26}H_{48}O_{17}P_4^{\bullet-}$<br>756.1848<br>756.1879	$C_{23}H_{41}O_{13}P_3^{\bullet-}$<br>618.1766<br>618.1795	$C_{18}H_{30}O_9P_2^{\bullet-}$<br>452.1371<br>452.1373	$C_{15}H_{23}O_5P^{\bullet-}$<br>314.1289<br>*n.d.*	y_i^{z-}<br>m/z_{th}<br>m/z_{exp}
	$C_{42}H_{81}O_{32}P_8^{\bullet 2-}$<br>672.6312<br>672.6305	$C_{39}H_{74}O_{28}P_7^{\bullet 2-}$<br>603.6271<br>603.6241	$C_{34}H_{64}O_{24}P_6^{\bullet-}$<br>1042.2219<br>1042.2274	$C_{29}H_{53}O_{20}P_5^{\bullet-}$<br>876.1824<br>876.1807	$C_{26}H_{46}O_{16}P_4^{\bullet-}$<br>738.1742<br>738.1714	$C_{23}H_{39}O_{12}P_3^{\bullet-}$<br>600.1660<br>600.1674	$C_{18}H_{28}O_8P_2^{\bullet-}$<br>434.1265<br>434.1267	$C_{15}H_{21}O_4P^{\bullet-}$<br>296.1183<br>*n.d.*	z_i^{z-}<br>m/z_{th}<br>m/z_{exp}

Supplementary Table 9. Accurate mass measurements of sequencing fragments in the CID spectrum recorded for the third block coding for "e" in the "PHew!" pentablock (Supplementary Fig. **7c**), using the $[B3-2H]^{2-}$ precursor ion ($C_{61}H_{113}N_2O_{37}P_9^{\bullet\bullet 2-}$, *m/z* 872.2336) as an internal standard. 0-bit: $C_3H_7O_4P$; 1-bit: $C_5H_{11}O_4P$. *n.e.*: not expected. *n.d.*: not detected.

a_i^{z-}	$C_{26}H_{43}N_2O_5P^{\bullet-}$	$C_{31}H_{54}N_2O_9P_2^{\bullet-}$	$C_{36}H_{65}N_2O_{13}P_3^{\bullet-}$	$C_{41}H_{76}N_2O_{17}P_4^{\bullet-}$	$C_{44}H_{83}N_2O_{21}P_5^{\bullet-}$	$C_{49}H_{94}N_2O_{25}P_6^{\bullet-}$	$C_{54}H_{105}N_2O_{29}P_7^{\bullet-}$	$C_{59}H_{115}N_2O_{33}P_8^{\bullet 2-}$	
m/z_{th}	494.2915	660.3310	826.3765	992.4100	1130.4182	1296.4577	1462.4972	813.7647	
m/z_{exp}	*n.d.*	*n.d.*	826.3813	992.4162	1130.4224	1296.4550	*n.d.*	813.7662	
b_i^{z-}	$C_{26}H_{45}N_2O_6P^{\bullet-}$	$C_{31}H_{56}N_2O_{10}P_2^{\bullet-}$	$C_{36}H_{67}N_2O_{14}P_3^{\bullet-}$	$C_{41}H_{78}N_2O_{18}P_4^{\bullet-}$	$C_{44}H_{85}N_2O_{22}P_5^{\bullet-}$	$C_{49}H_{96}N_2O_{26}P_6^{\bullet-}$	$C_{54}H_{107}N_2O_{30}P_7^{\bullet-}$	$C_{59}H_{117}N_2O_{34}P_8^{\bullet 2-}$	
m/z_{th}	512.3021	678.3416	844.3811	1010.4206	1148.4288	1314.4683	1480.5078	822.7700	
m/z_{exp}	512.3016	678.3441	844.3862	1010.4188	1148.4265	1314.4690	1480.5011	822.7772	
c_i^{z-}	$C_{26}H_{44}N_2O_8P_2^{\bullet-}$	$C_{31}H_{55}N_2O_{12}P_3^{\bullet-}$	$C_{36}H_{66}N_2O_{16}P_4^{\bullet-}$	$C_{41}H_{77}N_2O_{20}P_5^{\bullet-}$	$C_{44}H_{84}N_2O_{24}P_6^{\bullet-}$	$C_{49}H_{95}N_2O_{28}P_7^{\bullet-}$	$C_{54}H_{105}N_2O_{32}P_8^{\bullet 2-}$	$C_{59}H_{116}N_2O_{36}P_9^{\bullet 2-}$	
m/z_{th}	574.2578	740.2973	906.3368	1072.3763	1210.3845	1376.4240	770.7281	853.7479	
m/z_{exp}	574.2577	*n.d.*	906.3381	1072.3699	1210.3830	*n.d.*	770.7241	853.7493	
d_i^{z-}	$C_{26}H_{46}N_2O_9P_2^{\bullet-}$	$C_{31}H_{57}N_2O_{13}P_3^{\bullet-}$	$C_{36}H_{68}N_2O_{17}P_4^{\bullet-}$	$C_{41}H_{79}N_2O_{21}P_5^{\bullet-}$	$C_{44}H_{86}N_2O_{25}P_6^{\bullet-}$	$C_{49}H_{96}N_2O_{29}P_7^{\bullet 2-}$	$C_{54}H_{107}N_2O_{33}P_8^{\bullet 2-}$	$C_{59}H_{118}N_2O_{37}P_9^{\bullet 2-}$	
m/z_{th}	592.2684	758.3079	924.3474	1090.3869	1228.3951	696.7137	779.7334	862.7532	
m/z_{exp}	*n.d.*	758.3126	924.3467	1090.3927	1228.3922	696.7161	779.7338	*n.d.*	
$i \rightarrow$	*1*	*2*	*3*	*4*	*5*	*6*	*7*	*8*	
	**0**	**1**	**1**	**1**	**0**	**1**	**1**	**1**	
	8	*7*	*6*	*5*	*4*	*3*	*2*	*1*	$\leftarrow j$
	$C_{46}H_{92}O_{36}P_9^{\bullet 2-}$	$C_{43}H_{85}O_{32}P_8^{\bullet 2-}$	$C_{38}H_{74}O_{28}P_7^{\bullet 2-}$	$C_{33}H_{64}O_{24}P_6^{\bullet-}$	$C_{28}H_{53}O_{20}P_5^{\bullet-}$	$C_{25}H_{46}O_{16}P_4^{\bullet-}$	$C_{20}H_{35}O_{12}P_3^{\bullet-}$	$C_{15}H_{24}O_8P_2^{\bullet-}$	w_i^{z-}
	749.6509	680.6468	597.6271	1030.2219	864.1824	726.1742	560.1347	394.0952	m/z_{th}
	n.d.	680.6467	597.6282	1030.2240	864.1841	726.1697	560.1321	394.0955	m/z_{exp}
	$C_{46}H_{90}O_{35}P_9^{\bullet 2-}$	$C_{43}H_{83}O_{31}P_8^{\bullet 2-}$	$C_{38}H_{72}O_{27}P_7^{\bullet 2-}$	$C_{33}H_{62}O_{23}P_6^{\bullet-}$	$C_{28}H_{51}O_{19}P_5^{\bullet-}$	$C_{25}H_{44}O_{15}P_4^{\bullet-}$	$C_{20}H_{33}O_{11}P_3^{\bullet-}$	$C_{15}H_{22}O_7P_2^{\bullet-}$	x_i^{z-}
	740.6456	671.6415	588.6218	1012.2113	846.1718	708.1636	542.1241	376.0846	m/z_{th}
	n.d.	671.6427	588.6237	*n.d.*	846.1777	708.1675	542.1262	376.0850	m/z_{exp}
	$C_{46}H_{91}O_{33}P_8^{\bullet 2-}$	$C_{43}H_{85}O_{29}P_7^{\bullet-}$	$C_{38}H_{74}O_{25}P_6^{\bullet-}$	$C_{33}H_{63}O_{21}P_5^{\bullet-}$	$C_{28}H_{52}O_{17}P_4^{\bullet-}$	$C_{25}H_{45}O_{13}P_3^{\bullet-}$	$C_{20}H_{34}O_9P_2^{\bullet-}$	$C_{15}H_{23}O_5P^{\bullet-}$	y_i^{z-}
	709.6677	1282.3345	1116.2950	950.2556	784.2161	646.2079	480.1684	314.1289	m/z_{th}
	709.6727	1282.3394	1116.2910	950.2575	784.2175	646.2146	480.1680	*n.d.*	m/z_{exp}
	$C_{46}H_{89}O_{32}P_8^{\bullet 2-}$	$C_{43}H_{82}O_{28}P_7^{\bullet-}$	$C_{38}H_{72}O_{24}P_6^{\bullet-}$	$C_{33}H_{61}O_{20}P_5^{\bullet-}$	$C_{28}H_{50}O_{16}P_4^{\bullet-}$	$C_{25}H_{43}O_{12}P_3^{\bullet-}$	$C_{20}H_{32}O_8P_2^{\bullet-}$	$C_{15}H_{21}O_4P^{\bullet-}$	z_i^{z-}
	700.6625	1264.3240	1098.2845	932.2450	766.2055	628.1973	462.1578	296.1183	m/z_{th}
	700.6602	1264.3241	1098.2786	*n.d.*	766.2029	628.1927	462.1577	*n.d.*	m/z_{exp}

Supplementary Table 10. Accurate mass measurements of sequencing fragments in the CID spectrum recorded for the fourth block coding for "w" in the "PHew!" pentablock (Supplementary Fig. 7d), using the $[B4 - 2H]^{2-}$ precursor ion ($C_{69}H_{131}N_2O_{37}P_9^{\bullet\bullet 2-}$, *m/z* 928.2962) as an internal standard. 0-bit: $C_3H_7O_4P$; 1-bit: $C_5H_{11}O_4P$. *n.e.*: not expected. *n.d.*: not detected.

a_i^{z-}<br>m/z_{th}<br>m/z_{exp}	$C_{22}H_{35}N_2O_5P^{\bullet-}$<br>438.2289<br>438.2230	$C_{27}H_{46}N_2O_9P_2^{\bullet-}$<br>604.2684<br>604.2663	$C_{30}H_{53}N_2O_{13}P_3^{\bullet-}$<br>742.2766<br>742.2767	$C_{33}H_{60}N_2O_{17}P_4^{\bullet-}$<br>880.2848<br>880.2855	$C_{36}H_{67}N_2O_{21}P_5^{\bullet-}$<br>1018.2930<br>1018.2930	$C_{39}H_{74}N_2O_{25}P_6^{\bullet-}$<br>1156.3012<br>1156.2983	$C_{42}H_{81}N_2O_{29}P_7^{\bullet-}$<br>1294.3094<br>1294.3114	$C_{47}H_{91}N_2O_{33}P_8^{\bullet 2-}$<br>729.6708<br>729.6688	
b_i^{z-}<br>m/z_{th}<br>m/z_{exp}	$C_{22}H_{37}N_2O_6P^{\bullet-}$<br>456.2395<br>456.2379	$C_{27}H_{48}N_2O_{10}P_2^{\bullet-}$<br>622.2790<br>622.2828	$C_{30}H_{55}N_2O_{14}P_3^{\bullet-}$<br>760.2872<br>760.2885	$C_{33}H_{62}N_2O_{18}P_4^{\bullet-}$<br>898.2954<br>898.2994	$C_{36}H_{69}N_2O_{22}P_5^{\bullet-}$<br>1036.3036<br>1036.3047	$C_{39}H_{76}N_2O_{26}P_6^{\bullet-}$<br>1174.3118<br>1174.3138	$C_{42}H_{82}N_2O_{30}P_7^{\bullet 2-}$<br>655.6563<br>655.6597	*n.e.*	
c_i^{z-}<br>m/z_{th}<br>m/z_{exp}	$C_{22}H_{36}N_2O_8P_2^{\bullet-}$<br>518.1952<br>518.1999	$C_{27}H_{47}N_2O_{12}P_3^{\bullet-}$<br>684.2347<br>684.2366	$C_{30}H_{54}N_2O_{16}P_4^{\bullet-}$<br>822.2429<br>822.2408	$C_{33}H_{61}N_2O_{20}P_5^{\bullet-}$<br>960.2511<br>960.2507	$C_{36}H_{68}N_2O_{24}P_6^{\bullet-}$<br>1098.2593<br>1098.2657	$C_{39}H_{75}N_2O_{28}P_7^{\bullet-}$<br>1236.2675<br>1236.2692	$C_{42}H_{81}N_2O_{32}P_8^{\bullet 2-}$<br>686.6342<br>686.6390	*n.e.*	
d_i^{z-}<br>m/z_{th}<br>m/z_{exp}	$C_{22}H_{38}N_2O_9P_2^{\bullet-}$<br>536.2058<br>536.2080	$C_{27}H_{49}N_2O_{13}P_3^{\bullet-}$<br>702.2453<br>702.2466	$C_{30}H_{56}N_2O_{17}P_4^{\bullet-}$<br>840.2535<br>840.2579	$C_{33}H_{63}N_2O_{21}P_5^{\bullet-}$<br>978.2617<br>978.2600	$C_{36}H_{70}N_2O_{25}P_6^{\bullet-}$<br>1116.2699<br>1116.2709	$C_{39}H_{76}N_2O_{29}P_7^{\bullet 2-}$<br>626.6354<br>626.6355	$C_{42}H_{83}N_2O_{33}P_8^{\bullet 2-}$<br>695.6395<br>*n.d.*	*n.e.*	
$i \rightarrow$	*1*	*2*	*3*	*4*	*5*	*6*	*7*	*8*	
	**0**	**1**	**0**	**0**	**0**	**0**	**0**	**1**	
	8	*7*	*6*	*5*	*4*	*3*	*2*	*1*	$\leftarrow j$
	$C_{28}H_{64}O_{33}P_8^{2-}$<br>588.0621<br>*n.d.*	$C_{25}H_{57}O_{29}P_7^{2-}$<br>519.0580<br>519.0583	$C_{20}H_{47}O_{25}P_6^{-}$<br>873.0838<br>873.0854	$C_{17}H_{40}O_{21}P_5^{-}$<br>735.0756<br>735.0770	$C_{14}H_{33}O_{17}P_4^{-}$<br>597.0674<br>597.0685	$C_{11}H_{26}O_{13}P_3^{-}$<br>459.0592<br>459.0609	$C_8H_{19}O_9P_2^{-}$<br>321.0510<br>321.0523	$C_5H_{12}O_5P^{-}$<br>183.0428	w_i^{z-}<br>m/z_{th}<br>m/z_{exp}
	$C_{28}H_{62}O_{32}P_8^{2-}$<br>579.0568<br>579.0582	$C_{25}H_{55}O_{28}P_7^{2-}$<br>510.0527<br>510.0549	$C_{20}H_{45}O_{24}P_6^{-}$<br>855.0732<br>855.0754	$C_{17}H_{38}O_{20}P_5^{-}$<br>717.0850<br>717.0782	$C_{14}H_{31}O_{16}P_4^{-}$<br>579.0568<br>579.0582	$C_{11}H_{24}O_{12}P_3^{-}$<br>441.0486<br>441.0506	$C_8H_{17}O_8P_2^{-}$<br>303.0404<br>303.0417	*n.e.*	x_i^{z-}<br>m/z_{th}<br>m/z_{exp}
	$C_{28}H_{63}O_{30}P_7^{2-}$<br>548.0789<br>548.0817	$C_{25}H_{57}O_{26}P_6^{-}$<br>959.1569<br>959.1572	$C_{20}H_{46}O_{22}P_5^{-}$<br>793.1174<br>793.1208	$C_{17}H_{39}O_{18}P_4^{-}$<br>655.1092<br>655.1130	$C_{14}H_{32}O_{14}P_3^{-}$<br>517.1010<br>517.1019	$C_{11}H_{25}O_{10}P_2^{-}$<br>379.0928<br>379.0921	$C_8H_{18}O_6P^{-}$<br>241.0847<br>241.0890	*n.e.*	y_i^{z-}<br>m/z_{th}<br>m/z_{exp}
	$C_{28}H_{62}O_{29}P_7^{-}$<br>1079.1546<br>1079.1550	$C_{25}H_{55}O_{25}P_6^{-}$<br>941.1464<br>941.1459	$C_{20}H_{44}O_{21}P_5^{-}$<br>775.1069<br>775.1055	$C_{17}H_{37}O_{17}P_4^{-}$<br>637.0987<br>637.1002	$C_{14}H_{30}O_{13}P_3^{-}$<br>499.0905<br>499.0916	$C_{11}H_{23}O_9P_2^{-}$<br>361.0823<br>361.0834	$C_8H_{16}O_5P^{-}$<br>223.0741<br>223.0749	*n.e.*	z_i^{z-}<br>m/z_{th}<br>m/z_{exp}

Supplementary Table 11. Accurate mass measurements of sequencing fragments in the CID spectrum recorded for the last block coding for "!" in the "PHew!" pentablock (Supplementary Fig. 7e), using the $[B5 - 2H]^{2-}$ precursor ion ($C_{47}H_{93}N_2O_{34}P_8^{\bullet 2-}$, *m/z* 738.6761) as an internal standard. 0-bit: $C_3H_7O_4P$; 1-bit: $C_5H_{11}O_4P$. *n.e.*: not expected. *n.d.*: not detected.

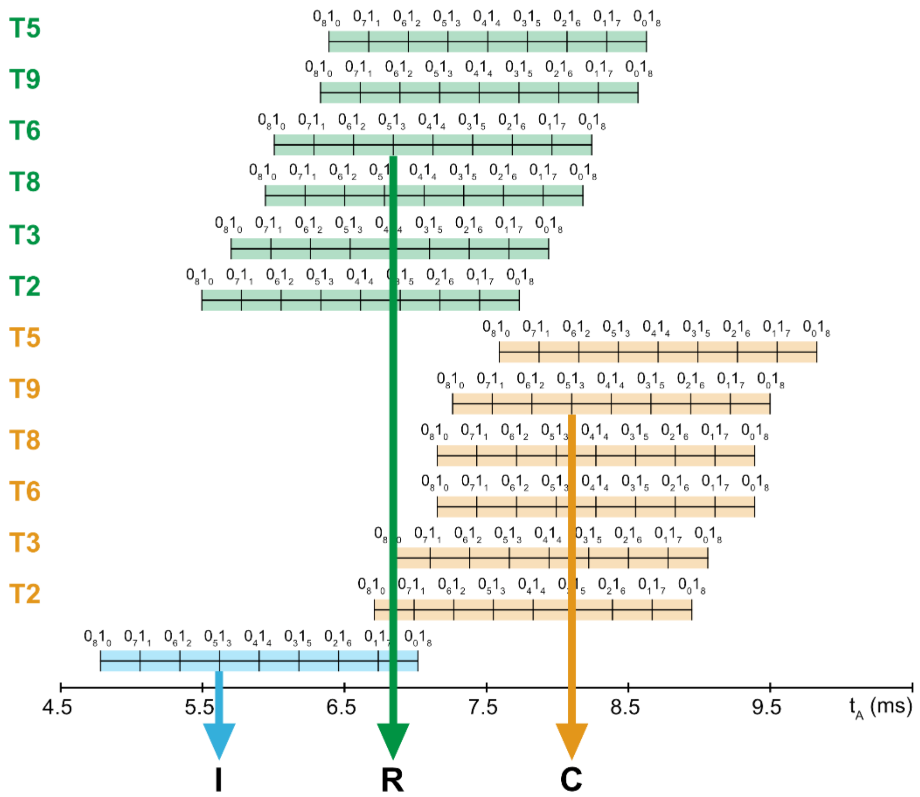

Supplementary Fig. 8. Predicting chart for selecting tags to label singly charged blocks. Predicted t_A ranges for singly deprotonated blocks $[Bi - H]^-$ generated in MALDI-MS as a function of their category (first: blue, inner: orange, last: green) and their Ti tag indicated on the left, using WV = 25 V and WH = 130 m.s^{-1}.

a_i^{z-}<br>m/z_{th}<br>m/z_{exp}	*n.e.*	*n.e.*	$C_{11}H_{23}O_9P_2^-$<br>361.0823<br>361.0764	$C_{14}H_{30}O_{13}P_3^-$<br>499.0905<br>499.0885	$C_{19}H_{41}O_{17}P_4^-$<br>665.1300<br>665.1379	$C_{22}H_{48}O_{21}P_5^-$<br>803.1382<br>803.1411	$C_{25}H_{55}O_{25}P_6^-$<br>941.1464<br>941.1474	$C_{30}H_{66}O_{29}P_7^-$<br>1107.1859<br>1107.1845	
b_i^{z-}<br>m/z_{th}<br>m/z_{exp}	*n.e.*	*n.e.*	$C_{11}H_{25}O_{10}P_2^-$<br>379.0928<br>379.0867	$C_{14}H_{32}O_{14}P_3^-$<br>517.1010<br>517.1007	$C_{19}H_{43}O_{18}P_4^-$<br>683.1405<br>683.1437	$C_{22}H_{50}O_{22}P_5^-$<br>821.1487<br>821.1478	$C_{25}H_{57}O_{26}P_6^-$<br>959.1569<br>959.1591	$C_{30}H_{68}O_{30}P_7^-$<br>1125.1964<br>1125.1942	
c_i^{z-}<br>m/z_{th}<br>m/z_{exp}	*n.e.*	$C_8H_{17}O_8P_2^-$<br>303.0404<br>303.0394	$C_{11}H_{24}O_{12}P_3^-$<br>441.0486<br>441.0446	$C_{14}H_{31}O_{16}P_4^-$<br>579.0568<br>579.0582	$C_{19}H_{42}O_{20}P_5^-$<br>745.0963<br>745.1028	$C_{22}H_{49}O_{24}P_6^-$<br>883.1045<br>883.1106	$C_{25}H_{56}O_{28}P_7^-$<br>1021.1127<br>1021.1147	$C_{30}H_{67}O_{32}P_8^-$<br>1187.1522<br>1187.1542	
d_i^{z-}<br>m/z_{th}<br>m/z_{exp}	$C_3H_8O_5P^-$<br>155.0114<br>*n.r.*	$C_8H_{19}O_9P_2^-$<br>321.0509<br>321.0518	$C_{11}H_{26}O_{13}P_3^-$<br>459.0591<br>459.0546	$C_{14}H_{33}O_{17}P_4^-$<br>597.0674<br>597.0684	$C_{19}H_{44}O_{21}P_5^{1-}$<br>763.1069<br>763.1110	$C_{22}H_{51}O_{25}P_6^-$<br>901.1151<br>901.1129	$C_{25}H_{58}O_{29}P_7^-$<br>1038.1233<br>1038.1260	$C_{30}H_{69}O_{33}P_8^-$<br>1205.1628<br>*n.d.*	
$i \rightarrow$	*1*	*2*	*3*	*4*	*5*	*6*	*7*	*8*	
	**0**	**1**	**0**	**0**	**1**	**0**	**0**	**1**	
	8	*7*	*6*	*5*	*4*	*3*	*2*	*1*	$\leftarrow j$
	n.e.	$C_{37}H_{74}O_{32}P_8^{\bullet-}$<br>1278.2070<br>1278.2026	$C_{32}H_{63}O_{28}P_7^{\bullet-}$<br>1112.1675<br>*n.d.*	$C_{29}H_{56}O_{24}P_6^{\bullet-}$<br>974.1593<br>974.1632	$C_{26}H_{49}O_{20}P_5^{\bullet-}$<br>836.1511<br>836.1472	$C_{21}H_{38}O_{16}P_4^{\bullet-}$<br>670.1116<br>*n.d.*	$C_{18}H_{31}O_{12}P_3^{\bullet-}$<br>532.1034<br>532.1033	$C_{15}H_{24}O_8P_2^{\bullet-}$<br>394.0952<br>394.0919	w_i^{z-}<br>m/z_{th}<br>m/z_{exp}
	n.e.	$C_{37}H_{72}O_{31}P_8^{\bullet-}$<br>1260.1964<br>*n.d.*	$C_{32}H_{61}O_{27}P_7^{\bullet-}$<br>1094.1569<br>*n.d.*	$C_{29}H_{54}O_{23}P_6^{\bullet-}$<br>956.1487<br>956.1481	$C_{26}H_{47}O_{19}P_5^{\bullet-}$<br>818.1405<br>818.1428	$C_{21}H_{36}O_{15}P_4^{\bullet-}$<br>652.1010<br>652.1056	$C_{18}H_{29}O_{11}P_3^{\bullet-}$<br>514.0928<br>514.0859	$C_{15}H_{22}O_7P_2^{\bullet-}$<br>376.0846<br>376.0768	x_i^{z-}<br>m/z_{th}<br>m/z_{exp}
	n.e.	$C_{37}H_{73}O_{29}P_7^{\bullet-}$<br>1198.2406<br>1198.2411	$C_{32}H_{62}O_{25}P_6^{\bullet-}$<br>1032.2011<br>1032.2028	$C_{29}H_{55}O_{21}P_5^{\bullet-}$<br>894.1930<br>894.1922	$C_{26}H_{48}O_{17}P_4^{\bullet-}$<br>756.1848<br>756.1899	$C_{21}H_{37}O_{13}P_3^{\bullet-}$<br>590.1453<br>590.1472	$C_{18}H_{30}O_9P_2^{\bullet-}$<br>452.1371<br>*n.d.*	$C_{15}H_{23}O_5P^{\bullet-}$<br>314.1289<br>314.1299	y_i^{z-}<br>m/z_{th}<br>m/z_{exp}
	$C_{40}H_{78}O_{32}P_8^{\bullet-}$<br>1318.2383<br>1318.2356	$C_{37}H_{71}O_{28}P_7^{\bullet-}$<br>1180.2301<br>1180.2360	$C_{32}H_{60}O_{24}P_6^{\bullet-}$<br>1014.1906<br>1014.1947	$C_{29}H_{53}O_{20}P_5^{\bullet-}$<br>876.1824<br>876.1843	$C_{26}H_{46}O_{16}P_4^{\bullet-}$<br>738.1742<br>738.1766	$C_{21}H_{35}O_{12}P_3^{\bullet-}$<br>572.1347<br>572.1397	$C_{18}H_{28}O_8P_2^{\bullet-}$<br>434.1265<br>434.1208	$C_{15}H_{21}O_4P^{\bullet-}$<br>296.1183<br>*n.r.*	z_i^{z-}<br>m/z_{th}<br>m/z_{exp}

Supplementary Table 12. Accurate mass measurements of sequencing fragments in the CID spectrum recorded for the first block coding for "I" in the "ICR" triblock (Fig. 6b), using the $[B1 - H]^-$ precursor ion ($C_{40}H_{80}O_{33}P_8^{\bullet-}$, *m/z* 1336.2488) formed in MALDI as an internal standard. 0-bit: $C_3H_7O_4P$; 1-bit: $C_5H_{11}O_4P$. *n.e.*: not expected. *n.d.*: not detected. *n.r.*: not recorded.

a_i^{z-}<br>m/z_{th}<br>m/z_{exp}	$C_{26}H_{43}N_2O_5P^{\bullet-}$<br>494.2915<br>*n.d.*	$C_{31}H_{54}N_2O_9P_2^{\bullet-}$<br>660.3310<br>*n.d.*	$C_{34}H_{61}N_2O_{13}P_3^{\bullet-}$<br>798.3392<br>798.3456	$C_{37}H_{68}N_2O_{17}P_4^{\bullet-}$<br>936.3474<br>936.3522	$C_{40}H_{75}N_2O_{21}P_5^{\bullet-}$<br>1074.3556<br>1074.3552	$C_{43}H_{82}N_2O_{25}P_6^{\bullet-}$<br>1212.3638<br>1212.3685	$C_{48}H_{93}N_2O_{29}P_7^{\bullet-}$<br>1378.4033<br>1378.4064	$C_{53}H_{104}N_2O_{33}P_8^{\bullet-}$<br>1544.4428<br>*n.d.*	
b_i^{z-}<br>m/z_{th}<br>m/z_{exp}	$C_{26}H_{45}N_2O_6P^{\bullet-}$<br>512.3021<br>*n.d.*	$C_{31}H_{56}N_2O_{10}P_2^{\bullet-}$<br>678.3416<br>*n.d.*	$C_{34}H_{63}N_2O_{14}P_3^{\bullet-}$<br>816.3498<br>816.3458	$C_{37}H_{70}N_2O_{18}P_4^{\bullet-}$<br>954.3580<br>954.3654	$C_{40}H_{77}N_2O_{22}P_5^{\bullet-}$<br>1092.3662<br>1092.3689	$C_{43}H_{84}N_2O_{26}P_6^{\bullet-}$<br>1230.3744<br>1230.3672	$C_{48}H_{95}N_2O_{30}P_7^{\bullet-}$<br>1396.4139<br>*n.d.*	$C_{53}H_{106}N_2O_{34}P_8^{\bullet-}$<br>1562.4534<br>1562.4471	
c_i^{z-}<br>m/z_{th}<br>m/z_{exp}	$C_{26}H_{44}N_2O_8P_2^{\bullet-}$<br>574.2578<br>*n.d.*	$C_{31}H_{55}N_2O_{12}P_3^{\bullet-}$<br>740.2973<br>740.3010	$C_{34}H_{62}N_2O_{16}P_4^{\bullet-}$<br>878.3055<br>878.3026	$C_{37}H_{69}N_2O_{20}P_5^{\bullet-}$<br>1016.3137<br>1016.3110	$C_{40}H_{76}N_2O_{24}P_6^{\bullet-}$<br>1154.3219<br>1154.3284	$C_{43}H_{83}N_2O_{28}P_7^{\bullet-}$<br>1292.3301<br>1292.3314	$C_{48}H_{94}N_2O_{32}P_8^{\bullet-}$<br>1458.3696<br>1458.3716	$C_{53}H_{105}N_2O_{36}P_9^{\bullet-}$<br>1624.4091<br>1624.4089	
d_i^{z-}<br>m/z_{th}<br>m/z_{exp}	$C_{26}H_{46}N_2O_9P_2^{\bullet-}$<br>592.2684<br>592.2679	$C_{31}H_{57}N_2O_{13}P_3^{\bullet-}$<br>758.3079<br>758.3157	$C_{34}H_{64}N_2O_{17}P_4^{\bullet-}$<br>896.3161<br>896.3135	$C_{37}H_{71}N_2O_{21}P_5^{\bullet-}$<br>1034.3244<br>1034.3226	$C_{40}H_{78}N_2O_{25}P_6^{\bullet-}$<br>1172.3325<br>1172.3372	$C_{43}H_{85}N_2O_{29}P_7^{\bullet-}$<br>1310.3407<br>1310.3390	$C_{48}H_{96}N_2O_{33}P_8^{\bullet-}$<br>1476.3802<br>*n.d.*	$C_{53}H_{107}N_2O_{37}P_9^{\bullet-}$<br>1642.4197<br>*n.d.*	
$i \rightarrow$	*1*	*2*	*3*	*4*	*5*	*6*	*7*	*8*	
	**0**	**1**	**0**	**0**	**0**	**0**	**1**	**1**	
	8	*7*	*6*	*5*	*4*	*3*	*2*	*1*	$\leftarrow j$
	$C_{40}H_{81}O_{36}P_9^{\bullet-}$<br>1416.2152<br>1416.2190	$C_{37}H_{74}O_{32}P_8^{\bullet-}$<br>1278.2070<br>1278.2148	$C_{32}H_{63}O_{28}P_7^{\bullet-}$<br>1112.1675<br>*n.d.*	$C_{29}H_{56}O_{24}P_6^{\bullet-}$<br>974.1593<br>974.1607	$C_{26}H_{49}O_{20}P_5^{\bullet-}$<br>836.1511<br>836.1568	$C_{23}H_{42}O_{16}P_4^{\bullet-}$<br>698.1429<br>698.1373	$C_{20}H_{35}O_{12}P_3^{\bullet-}$<br>560.1347<br>560.1387	$C_{15}H_{24}O_8P_2^{\bullet-}$<br>394.0952<br>394.0978	w_i^{z-}<br>m/z_{th}<br>m/z_{exp}
	$C_{40}H_{79}O_{35}P_9^{\bullet-}$<br>1398.2046<br>1398.2084	$C_{37}H_{72}O_{31}P_8^{\bullet-}$<br>1260.1964<br>*n.d.*	$C_{32}H_{61}O_{27}P_7^{\bullet-}$<br>1094.1569<br>*n.d.*	$C_{29}H_{54}O_{23}P_6^{\bullet-}$<br>956.1487<br>956.1437	$C_{26}H_{47}O_{19}P_5^{\bullet-}$<br>818.1405<br>818.1407	$C_{23}H_{40}O_{15}P_4^{\bullet-}$<br>680.1323<br>680.1309	$C_{20}H_{33}O_{11}P_3^{\bullet-}$<br>542.1241<br>542.1279	$C_{15}H_{22}O_7P_2^{\bullet-}$<br>376.0846<br>*n.d.*	x_i^{z-}<br>m/z_{th}<br>m/z_{exp}
	$C_{40}H_{80}O_{33}P_8^{\bullet-}$<br>1336.2488<br>1336.2546	$C_{37}H_{73}O_{29}P_7^{\bullet-}$<br>1198.2406<br>1198.2461	$C_{32}H_{62}O_{25}P_6^{\bullet-}$<br>1032.2011<br>1032.2042	$C_{29}H_{55}O_{21}P_5^{\bullet-}$<br>894.1930<br>894.1892	$C_{26}H_{48}O_{17}P_4^{\bullet-}$<br>756.1848<br>*n.d.*	$C_{23}H_{41}O_{13}P_3^{\bullet-}$<br>618.1766<br>618.1715	$C_{20}H_{34}O_9P_2^{\bullet-}$<br>480.1684<br>480.1641	$C_{15}H_{23}O_5P^{\bullet-}$<br>314.1289<br>*n.d.*	y_i^{z-}<br>m/z_{th}<br>m/z_{exp}
	$C_{40}H_{78}O_{32}P_8^{\bullet-}$<br>1318.2383<br>1318.2361	$C_{37}H_{71}O_{28}P_7^{\bullet-}$<br>1180.2301<br>*n.d.*	$C_{32}H_{60}O_{24}P_6^{\bullet-}$<br>1014.1906<br>1014.2001	$C_{29}H_{53}O_{20}P_5^{\bullet-}$<br>876.1824<br>876.1779	$C_{26}H_{46}O_{16}P_4^{\bullet-}$<br>738.1742<br>738.1772	$C_{23}H_{39}O_{12}P_3^{\bullet-}$<br>600.1660<br>600.1585	$C_{20}H_{32}O_8P_2^{\bullet-}$<br>462.1578<br>*n.d.*	$C_{15}H_{21}O_4P^{\bullet-}$<br>296.1183<br>*n.r.*	z_i^{z-}<br>m/z_{th}<br>m/z_{exp}

Supplementary Table 13. Accurate mass measurements of sequencing fragments in the CID spectrum recorded for the second block coding for "C" in the "ICR" triblock (Fig. 6c), using the $[B2 - H]^-$ precursor ion ($C_{63}H_{118}N_2O_{37}P_9^{\bullet\bullet-}$, *m/z* 1773.5058) formed in MALDI as an internal standard. 0-bit: $C_3H_7O_4P$; 1-bit: $C_5H_{11}O_4P$. *n.e.*: not expected. *n.d.*: not detected. *n.r.*: not recorded.

a_i^{z-}<br>m/z_{th}<br>m/z_{exp}	$C_{26}H_{37}N_2O_5P^{\bullet-}$<br>488.2446<br>*n.d.*	$C_{31}H_{48}N_2O_9P_2^{\bullet-}$<br>654.2841<br>*n.d.*	$C_{34}H_{55}N_2O_{13}P_3^{\bullet-}$<br>792.2923<br>792.2958	$C_{39}H_{66}N_2O_{17}P_4^{\bullet-}$<br>958.3318<br>958.3316	$C_{42}H_{73}N_2O_{21}P_5^{\bullet-}$<br>1096.3399<br>1096.3361	$C_{45}H_{80}N_2O_{25}P_6^{\bullet-}$<br>1234.3481<br>1234.3484	$C_{50}H_{91}N_2O_{29}P_7^{\bullet-}$<br>1400.3878<br>*n.d.*	$C_{53}H_{98}N_2O_{33}P_8^{\bullet-}$<br>1538.3958<br>*n.d.*	
b_i^{z-}<br>m/z_{th}<br>m/z_{exp}	$C_{26}H_{39}N_2O_6P^{\bullet-}$<br>506.2551<br>506.2608	$C_{31}H_{50}N_2O_{10}P_2^{\bullet-}$<br>672.2946<br>672.2926	$C_{34}H_{57}N_2O_{14}P_3^{\bullet-}$<br>810.3028<br>810.3030	$C_{39}H_{68}N_2O_{18}P_4^{\bullet-}$<br>976.3423<br>976.3344	$C_{42}H_{75}N_2O_{22}P_5^{\bullet-}$<br>1114.3505<br>1114.3483	$C_{45}H_{82}N_2O_{26}P_6^{\bullet-}$<br>1252.3587<br>1252.3616	$C_{50}H_{93}N_2O_{30}P_7^{\bullet-}$<br>1418.3982<br>1418.4031	*n.e.*	
c_i^{z-}<br>m/z_{th}<br>m/z_{exp}	$C_{26}H_{38}N_2O_8P_2^{\bullet-}$<br>568.2109<br>568.2063	$C_{31}H_{49}N_2O_{12}P_3^{\bullet-}$<br>734.2504<br>734.2512	$C_{34}H_{56}N_2O_{16}P_4^{\bullet-}$<br>872.2586<br>872.2554	$C_{39}H_{67}N_2O_{20}P_5^{\bullet-}$<br>1038.2981<br>1038.2973	$C_{42}H_{74}N_2O_{24}P_6^{\bullet-}$<br>1176.3063<br>1176.3040	$C_{45}H_{81}N_2O_{28}P_7^{\bullet-}$<br>1314.3145<br>1314.3118	$C_{50}H_{92}N_2O_{32}P_8^{\bullet-}$<br>1480.3540<br>1480.3470	*n.e.*	
d_i^{z-}<br>m/z_{th}<br>m/z_{exp}	$C_{26}H_{40}N_2O_9P_2^{\bullet-}$<br>586.2215<br>586.2156	$C_{31}H_{51}N_2O_{13}P_3^{\bullet-}$<br>752.2610<br>752.2623	$C_{34}H_{58}N_2O_{17}P_4^{\bullet-}$<br>890.2692<br>890.2600	$C_{39}H_{69}N_2O_{21}P_5^{\bullet-}$<br>1056.3086<br>1056.3123	$C_{42}H_{76}N_2O_{25}P_6^{\bullet-}$<br>1194.3168<br>1194.3185	$C_{45}H_{83}N_2O_{29}P_7^{\bullet-}$<br>1332.3250<br>*n.d.*	$C_{50}H_{94}N_2O_{33}P_8^{\bullet-}$<br>1498.3645<br>*n.d.*	*n.e.*	
$i \rightarrow$	*1*	*2*	*3*	*4*	*5*	*6*	*7*	*8*	
	**0**	**1**	**0**	**1**	**0**	**0**	**1**	**0**	
	8	*7*	*6*	*5*	*4*	*3*	*2*	*1*	$\leftarrow j$
	$C_{30}H_{69}O_{33}P_8^-$<br>1205.1628<br>1205.1675	$C_{27}H_{62}O_{29}P_7^-$<br>1067.1546<br>1067.1553	$C_{22}H_{51}O_{25}P_6^-$<br>901.1151<br>901.1204	$C_{19}H_{44}O_{21}P_5^-$<br>763.1069<br>763.1119	$C_{14}H_{33}O_{17}P_4^-$<br>597.0674<br>597.0698	$C_{11}H_{26}O_{13}P_3^-$<br>459.0592<br>459.0547	$C_8H_{19}O_9P_2^-$<br>321.0510<br>321.0503	$C_3H_8O_5P^-$<br>155.0115<br>*n.r.*	w_i^{z-}<br>m/z_{th}<br>m/z_{exp}
	$C_{30}H_{67}O_{32}P_8^-$<br>1187.1522<br>1187.1467	$C_{27}H_{60}O_{28}P_7^-$<br>1049.1440<br>1049.1506	$C_{22}H_{49}O_{24}P_6^-$<br>883.1045<br>883.0997	$C_{19}H_{42}O_{20}P_5^-$<br>745.0963<br>745.0966	$C_{14}H_{31}O_{16}P_4^-$<br>579.0568<br>579.0600	$C_{11}H_{24}O_{12}P_3^-$<br>441.0486<br>441.0443	$C_8H_{17}O_8P_2^-$<br>303.0404<br>303.0394	*n.e.*	x_i^{z-}<br>m/z_{th}<br>m/z_{exp}
	$C_{30}H_{68}O_{30}P_7^-$<br>1125.1964<br>1125.2047	$C_{27}H_{61}O_{26}P_6^-$<br>987.1882<br>987.1910	$C_{22}H_{50}O_{22}P_5^-$<br>821.1487<br>821.1487	$C_{19}H_{43}O_{18}P_4^-$<br>683.1405<br>683.1392	$C_{14}H_{32}O_{14}P_3^-$<br>517.1010<br>517.0975	$C_{11}H_{25}O_{10}P_2^-$<br>379.0928<br>379.0853	$C_8H_{18}O_6P^-$<br>241.0847<br>*n.r.*	*n.e.*	y_i^{z-}<br>m/z_{th}<br>m/z_{exp}
	$C_{30}H_{66}O_{29}P_7^-$<br>1107.1859<br>1107.1915	$C_{27}H_{59}O_{25}P_6^-$<br>969.1777<br>969.1732	$C_{22}H_{48}O_{21}P_5^-$<br>803.1382<br>803.1395	$C_{19}H_{41}O_{17}P_4^-$<br>665.1300<br>665.1232	$C_{14}H_{30}O_{13}P_3^-$<br>499.0905<br>499.0898	$C_{11}H_{23}O_9P_2^-$<br>361.0823<br>361.0761	$C_8H_{16}O_5P^-$<br>223.0741<br>*n.r.*	*n.e.*	z_i^{z-}<br>m/z_{th}<br>m/z_{exp}

Supplementary Table 14. Accurate mass measurements of sequencing fragments in the CID spectrum recorded for the last block coding for "R" in the "ICR" triblock (Fig. 6d), using the $[B3 - H]^-$ precursor ion ($C_{53}H_{99}N_2O_{34}P_8^{\bullet-}$, *m/z* 1556.4064) formed in MALDI as an internal standard. 0-bit: $C_3H_7O_4P$; 1-bit: $C_5H_{11}O_4P$. *n.e.*: not expected. *n.d.*: not detected. *n.r.*: not recorded.

Supplementary Protocol. De novo sequencing (case of unknown samples)

Choice of the primary precursor ion – As mentioned in the caption of Supplementary Fig. 1a, ESI mass spectra of b-PPDEs show a charge state distribution with the most abundant species always at a charge state equal to 3n – 2, with n the number of blocks in the polymer. Deciphering this charge state from the isotopic pattern of the major peak observed in MS permits to find the number of blocks and thus to define which precursor ion has to be selected for the first CID step, based on the rule "2 charges per block".

Reconstruction of the PDE block sequence (using data from Supplementary Fig. 1b, reproduced below for the sake of simplicity)

This step consists of using fragments containing multiple blocks (here diblock fragments at the 4– charge state, annotated in red) as regions of overlap to identify each Bi block amongst doubly charged fragments (annotated in blue) and so properly reconstruct the whole polymer sequence.

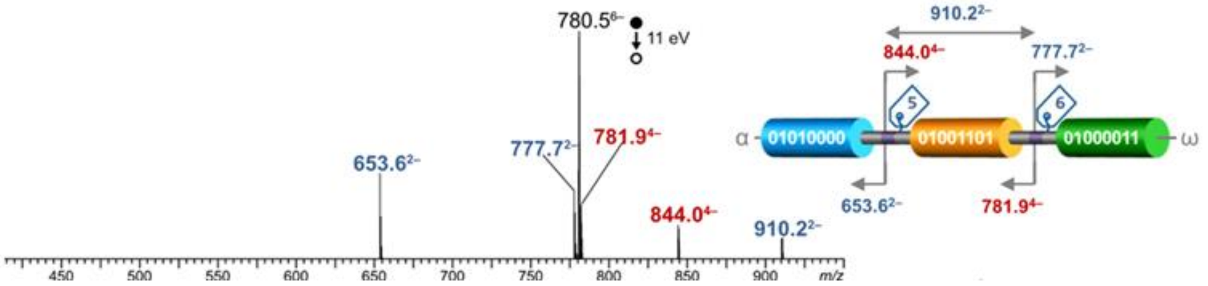

This is done by considering the following mathematical relationships:

Eq. 1: m(B1) + m(B2B3) = m(P) Eq. 2: m(B1) + m(B2) = m(B1B2)

Eq. 3: m(B1B2) + m(B3) = m(P) Eq. 4: m(B2) + m(B3) = m(B2B3)

where m(Bi) is the mass of the doubly charged Bi block obtained as 2 x *m/z*(Bi), m(BiBj) is the mass of the quadruply charged BiBj diblock obtained as 4 x *m/z*(BiBj), and m(P) is the mass of the precursor ion (here, $[P – 6H]^{6–}$ at *m/z* 780.5 so m(P) = 6 x 780.5 = 4683.0).

As mentioned in the main text, the first block is not labeled with any tag and is thus always the lightest one → amongst doubly charged fragments:

***m/z*(B1) = 653.6** so m(B1) = 1307.2

Knowing both m(P) and m(B1) permits to solve Eq. 1 for m(B2B3):

m(B2B3) = 4683.0 – 1307.2 = 3375.8

→ amongst quadruply charged diblocks annotated in red: B2B3 is at *m/z* = 3375.8/4 = 844.0.
The second 4– fragment at *m/z* 781.9 is necessarily B1B2, so m(B1B2) = 4 x 781.9 = 3127.6.
Knowing both m(B1) and m(B1B2) permits to solve Eq. 2 for m(B2):

m(B2) = 3127.6 – 1307.2 = 1820.4

→ amongst doubly charged fragments: ***m/z*(B2) = 910.2**

Accordingly, the last doubly charged fragment is necessarily B3: ***m/z*(B3) = 777.7**, which can be verified by calculating m(B3) either from Eq. 3: m(B3) = 4683.0 – 3127.6 = 1555.4 or from Eq. 4: m(B3) = 3375.8 – 1820.4 = 1555.4.

The same approach is used for MALDI data but in a much simpler way since all fragments (blocks and diblocks) are singly charged.

In the serial sequencing methodology involving IMS separation of blocks, the analytical output is a series of CID spectra extracted at t_A, still showing each secondary precursor (i.e. doubly charged blocks). From the rank of each block determined using the calculation described above and the measured t_A, the predicting chart (Fig. 4a for ESI, Supplementary Fig. 8 for MALDI) will permit to discover which tags were used to label each block as well as the block co-monomeric composition (the reader does not need to know this in advance). Finally, the binary sequence of each block is deciphered from CID data using the sequencing rules described from b-PPDEs (see reference in the main text).
